# Biomimetic Ru‐Mn Nanozyme with Cascade Catalytic Activity Attenuates Secondary Brain Injury in Intracerebral Hemorrhage

**DOI:** 10.1002/advs.202519340

**Published:** 2026-03-13

**Authors:** Zhongxin Duan, Peng Li, Yue Wang, Xin Qi, Wanyu Wang, Yongzhong Cheng, Huabin Zhang, Xin Hu, Xiang Gao

**Affiliations:** ^1^ Department of Neurosurgery and Institute of Neurosurgical Research State Key Laboratory of Biotherapy West China Medical School West China Hospital, Sichuan University Chengdu China; ^2^ State Key Laboratory of Metabolic Dysregulation & Prevention and Treatment of Esophageal Cancer Tianjian Laboratory of Advanced Biomedical Sciences School of Convergence Medicine Zhengzhou University Zhengzhou China; ^3^ Department of Neurosurgery and Neurosurgical Disease Research Centre the Second Affiliated Hospital of Guangzhou Medical University Guangzhou Medical University Guangzhou China

**Keywords:** intracerebral hemorrhage, oxidative stress, nanozyme, neuroinflammation, Ru‐Mn composite

## Abstract

Intracerebral hemorrhage (ICH) is a lethal stroke subtype with limited treatment options, largely due to secondary injury driven by oxidative stress and neuroinflammation. Here, we developed a ruthenium (Ru)‐manganese (Mn) composite nanozyme by integrating Ru nanozyme into Mn‐doped zeolitic imidazolate framework (Mn‐ZIF) to enable cascade catalytic activity for efficient ROS clearance. In vitro, Ru@Mn‐ZIF nanozyme reduced LPS‐induced microglia activation and H_2_O_2_‐induced neuronal oxidative damage, showing stronger protective effects than Mn‐ZIF nanozyme. In vivo, both intranasal and intravenous administration of Ru@Mn‐ZIF nanozyme significantly decreased hematoma volume, preserved blood‐brain barrier integrity, suppressed inflammatory responses, and improved neurological recovery in collagenase‐ and autologous blood‐induced ICH models. Biosafety evaluation revealed no pathological or biochemical abnormalities after treatment. These findings highlight Ru@Mn‐ZIF nanozyme as a promising therapeutic strategy for mitigating secondary brain injury and improving outcomes after ICH.

## Graphical abstract

Ru–Mn composite nanozymes are engineered to mimic dual‐enzyme cascade catalysis, enabling efficient ROS clearance and neuroinflammation suppression. In collagenase‐ and autologous blood‐induced ICH mouse models, intranasal and intravenous administration reduced hematoma volume, preserved BBB integrity, and improved neurological recovery. This work highlights Ru@Mn‐ZIF nanozyme as a promising therapeutic strategy with translational potential for ICH.

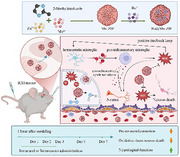

## 1 Introduction

Intracerebral hemorrhage (ICH) is a severe subtype of stroke characterized by non‐traumatic bleeding within the brain parenchyma [1]. Based on etiology, ICH can be classified into primary and secondary forms, with primary ICH accounting for 80–85% of cases, most commonly caused by hypertensive arteriolosclerosis and cerebral amyloid angiopathy [2]. Globally, ICH represents approximately 20% of the nearly 20 million new stroke cases annually [3]. Moreover, the high mortality rate and poor prognosis of ICH impose a substantial burden on both society and patients’ families. Owing to the complex pathophysiology of ICH, the development of effective strategies to improve functional recovery remains a significant challenge.

The pathophysiology of ICH involves the initial mechanical damage caused by hematoma formation and a cascade of secondary injuries [4]. Evolving from hours to days post‐ICH, secondary injuries are triggered by erythrocyte lysis, neuroinflammatory activation, and cytokine release, which collectively induce neuroinflammation, oxidative stress, BBB disruption, brain edema, and neuronal damage [5, 6]. Here, oxidative stress stands out as a core mechanism of ICH‐induced secondary brain injury. The brain's high lipid content, elevated iron levels, and limited antioxidant capacity render it particularly susceptible to oxidative stress. Critically, excessive oxidative stress amplifies the initial damage by intensifying neuroinflammation, promoting neuronal death, and impairing BBB integrity, forming a vicious cycle of secondary brain injury [7, 8]. Therefore, investigating the mechanisms and interventions for oxidative stress following ICH represents a promising therapeutic direction [9, 10].

Nanozymes, an emerging class of biomimetic nanomaterials, have gained significant attention for their therapeutic potential in neurological disorders [11]. These artificial enzymes are designed to structurally and functionally mimic natural oxidoreductases, such as superoxide dismutase (SOD), catalase (CAT), peroxidase (POD), and oxidase (OXD), offering high stability, sustained catalytic activity, and multifunctional enzyme‐like properties [12, 13]. Generally, nanozymes exhibiting SOD‐like or CAT‐like activity function to scavenge reactive oxygen species (ROS), whereas those with POD‐like or OXD‐like activity promote ROS generation [14, 15]. Recently, manganese (Mn)‐based, Ceria‐based, and ruthenium (Ru)‐based nanozymes with SOD‐like or CAT‐like activity have been demonstrated to effectively mitigate oxidative stress through ROS clearance [16, 17, 18]. Advancing this approach, bimetallic nanozymes offer a more promising therapeutic strategy by addressing key limitations of monometallic systems, including insufficient catalytic efficiency and singular enzymatic activity. Moreover, bimetallic nanozymes offer superior stability, broader environmental adaptability, and programmable theranostic functions, positioning them as a robust platform for effective intervention in neurological disorders [19, 20].

**SCHEME 1:**
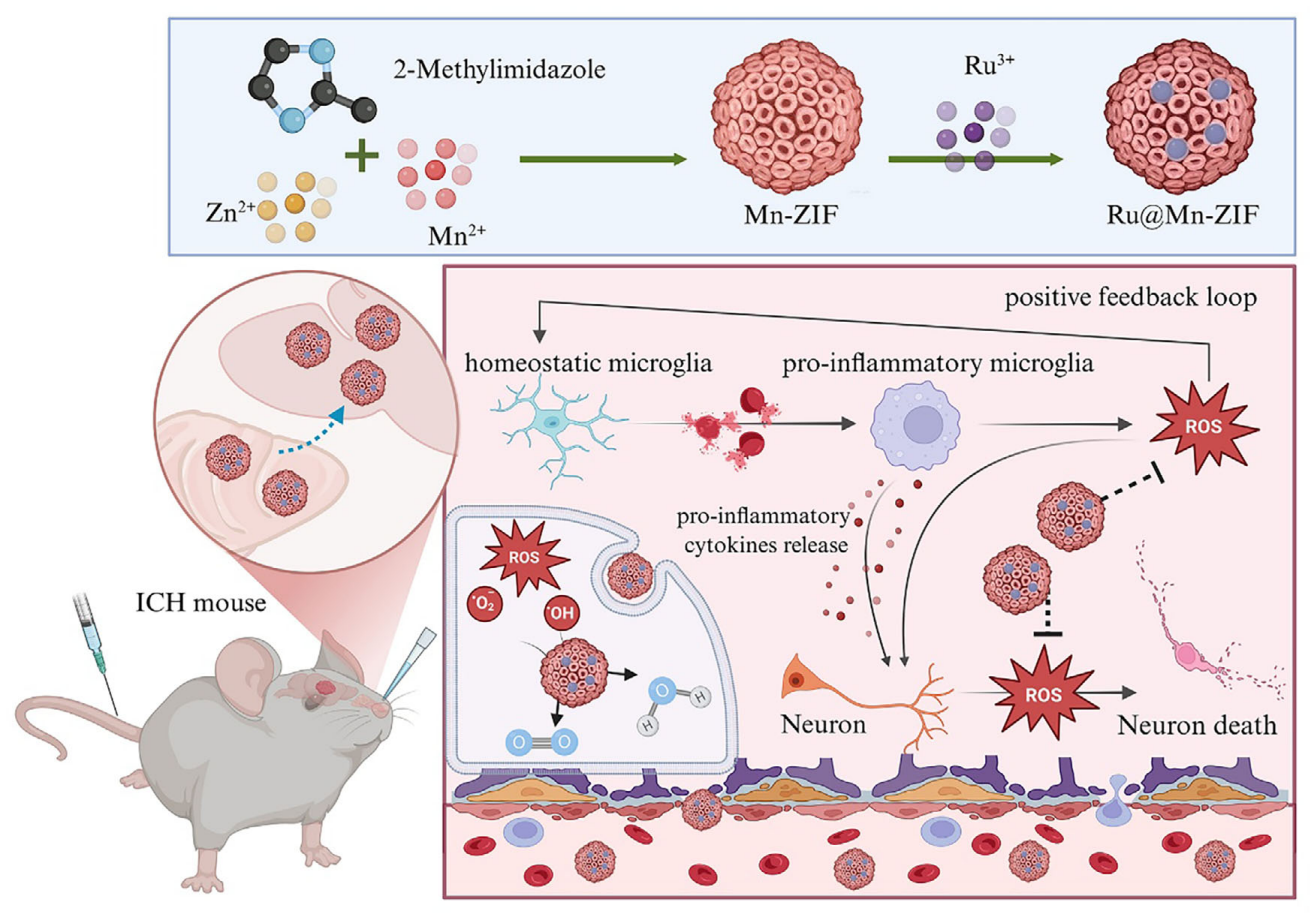
Nanozyme administration reduces neuronal death after ICH via scavenging ROS and suppressing microglia‐mediated pro‐neuroinflammation.

In this study, we developed a novel biomimetic Mn‐doped zeolitic imidazolate framework (Mn‐ZIF) nanozyme inspired by natural Mn‐SOD. To overcome the limitation of single enzymatic activity, Ru nanozyme was subsequently incorporated into the porous framework to achieve cascade catalysis. This cascade allowed complete conversion of hydrogen peroxide into oxygen and water, markedly enhancing free radical scavenging capacity. Benefiting from this design, the novel composite Ru@Mn‐ZIF nanozyme significantly promotes ROS clearance after ICH, modulates microglia‐mediated neuroinflammatory responses, and consequently alleviates secondary brain injury, ultimately improving neurological function (Scheme 1). Collectively, these findings showed the Ru@Mn‐ZIF nanozyme as a promising therapeutic agent for ICH.

## 2 Results and Discussion

### 2.1 Construction and Characterization of Ru@Mn‐ZIF Nanozyme

**FIGURE 1:**
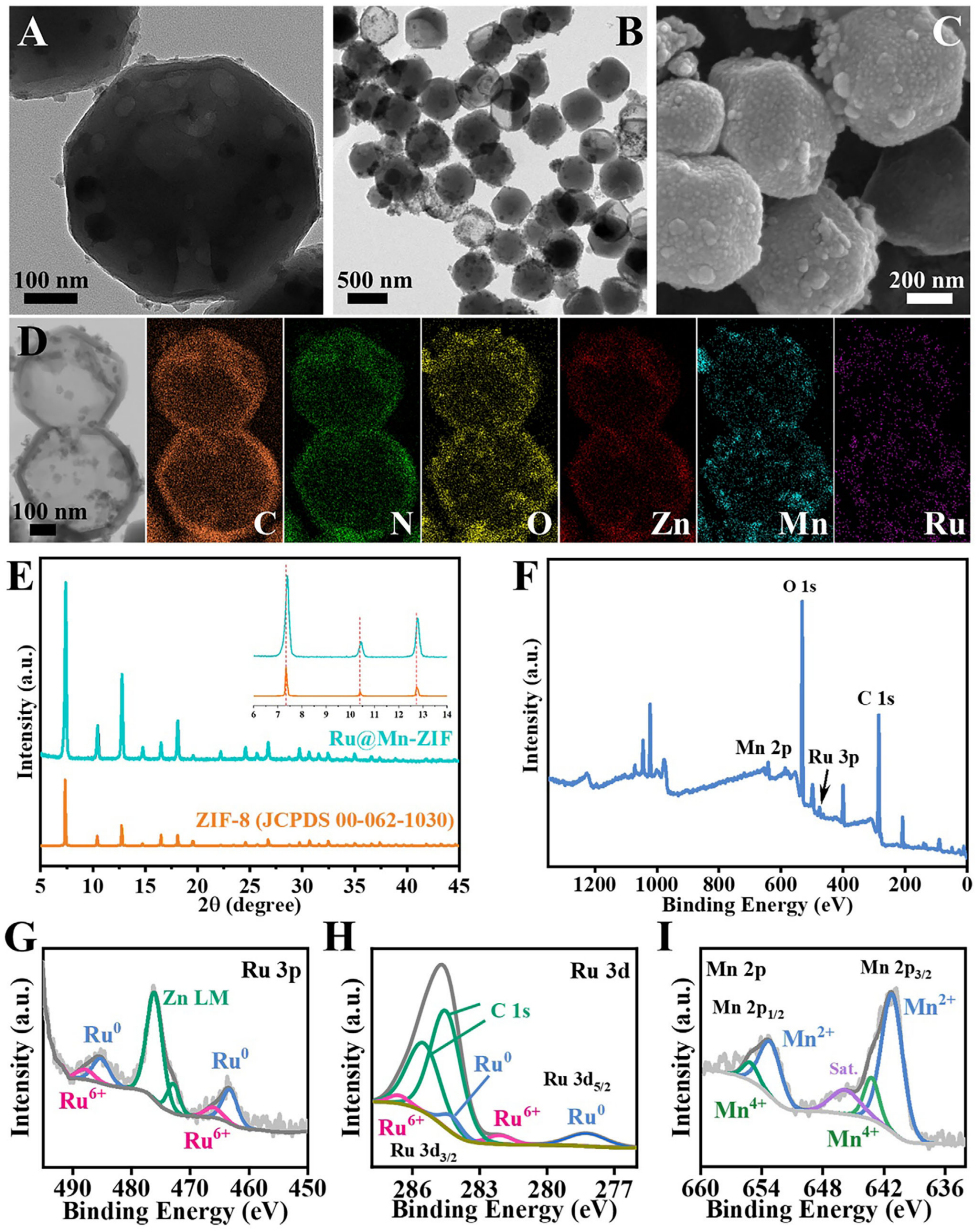
Characterization of Ru@Mn‐ZIF nanoparticles. The A, B) TEM, and C) SEM images of the as‐assembled Ru@Mn‐ZIF nanoparticles. (D) The elemental mapping analyses of the nanostructures correspond to the TEM image. (E) The PXRD patterns of the standard ZIF‐8 and the as‐fabricated Ru@Mn‐ZIF nanoparticles, and the inset in panel E represents the PXRD patterns of the corresponding species enlarged in a diffraction peak range of 2θ = 6–14° to clearly illustrate the shift of the main diffraction peaks of our nanocomposites. The XPS spectra of (F) all elements, (G) Ru 3p, (H) Ru 3d, and (I) Mn 2p of Ru@Mn‐ZIF nanoparticles, respectively.

The transmission electron microscopy (TEM) images of the as‐manufactured solids manifest themselves as a uniform hollow octahedral configuration with an average size of 450 nm, as shown in Figure 1A,B. In addition, numerous small fragments, which may be Ru(0) nanoparticles, were observed surrounding Ru@Mn‐ZIF. Furthermore, the scanning electron microscopy (SEM) images and elemental mapping analyses (Figure 1C,D) confirmed the presence of Ru(0) nanoparticles. The higher C content element was due to the TEM images taken by the carbon support membranes.

The crystal phase of the as‐prepared Ru@Mn‐ZIF samples was investigated by powder X‐ray diffraction (PXRD). As illustrated in Figure 1E, the diffraction pattern of the samples matches well with that of the standard ZIF‐8 (JCPDS no. 00‐062‐1030), consistent with those reported in previous literature, indicating a negligible impact of Ru on the framework structure of ZIF‐8. Significantly, the enlarged PXRD pattern indicates that compared to those of the standard ZIF‐8, the peaks of the main diffractions detected from our nanostructures exhibit an obvious shift to higher angles. This could be owing to the comparatively smaller ionic radius of Mn^2+^ (0.061 nm) than that of Zn^2+^ (0.074 nm). Meanwhile, this confirms that Mn^2+^ ions enter the interstitial sites of ZIF‐8 successfully. Unfortunately, due to the small particle size or low content of Ru(0), the identifiable diffraction peaks of metallic Ru(0) cannot be observed within the detection limit range.

The nature of the surface chemical environment and the bonding structure of the as‐fabricated Ru@Mn‐ZIF nanostructures were investigated via X‐ray photoelectron spectroscopy (XPS) spectra shown in Figure 1F–I. As presented in Figure 1G, two binding energy bands centered at ≈463.6 eV and 485.5 eV, which could be attributed to Ru 3p. These two bands could be deconvoluted to give out two sets of binding energy bands located at ≈463.2 eV, 466.1 eV and 485.1 eV, 487.8 eV, respectively, wherein those at ≈463.2 eV and 485.1 eV could be ascribed to the Ru° cations, while those at ≈466.1 and 487.8 eV could be attributed to metallic Ru^6+^ species. At the same time, the binding energy bands at ≈276.0 eV to 287.6 eV, which could be designated to Ru 3d_5/2_ and Ru 3d_3/2_, respectively, could be observed from the Ru 3d XPS spectra of the samples, as depicted in Figure 1H. A semi‐quantitative estimation of the XPS results of the samples (Figure 1G,H) indicates that the molar ratio between Ru^0^ and Ru^6+^ is ≈7:3. In addition, two bands at ≈640.9 eV and 653.4 eV are ascribed to Mn 2p_3/2_ and Mn 2p_1/2_, respectively (Figure 1I). A deconvolution of these bands gives out two sets of binding energies at ≈641.2 eV, 643.2 eV and ≈653.2 eV, 655.1 eV, respectively. The deconvoluted bands at ≈641.2 eV and 653.2 eV can be assigned to the Mn^2+^ cations, whereas those located at ≈643.2 eV and 655.1 eV can be assigned to Mn^4+^. A semi‐quantitative evaluation implies that the molar ratio of Mn^2+^ to Mn^4+^ is about 4:1. Taking into consideration the results of the SEM, TEM, elemental mapping analyses, PXRD, and XPS, it confirms the successful preparation of Ru@Mn‐ZIF nanostructures.

### 2.2 Enzyme‐Like Activity of Ru@Mn‐ZIF Nanozyme

**FIGURE 2:**
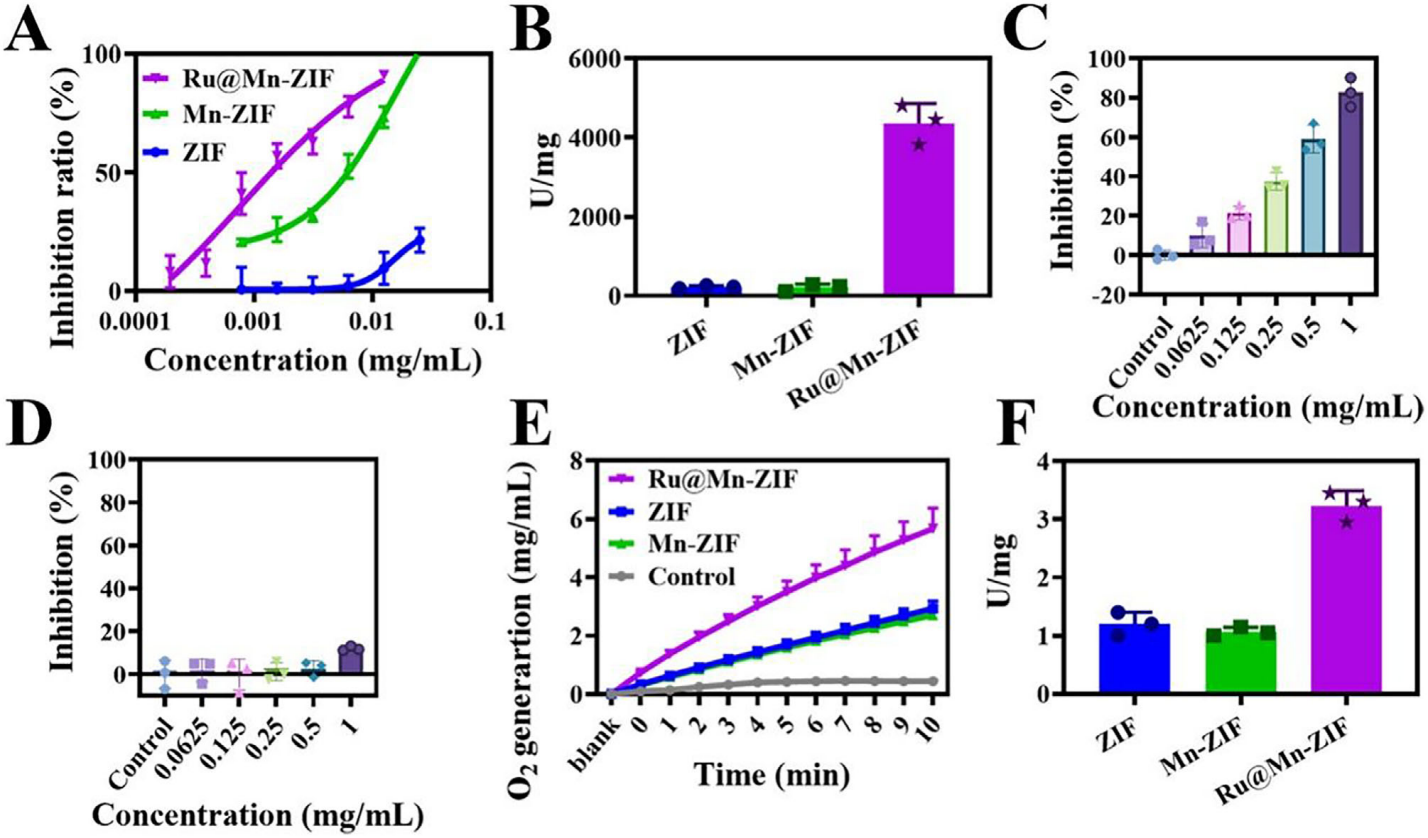
Enzyme‐like activity of Ru@Mn‐ZIF nanozyme. (A, B) The SOD‐like activities of Ru@Mn‐ZIF nanozyme. (C) DPPH as a substrate to detect nitrogen radical inhibition by Ru@Mn‐ZIF nanozyme. (D) ABTS as a substrate to detect nitrogen radical inhibition by Ru@Mn‐ZIF nanozyme. (E, F) The CAT‐like activities of Ru@Mn‐ZIF nanozyme. *n* = 3.

Following the successful synthesis of the nanomotors, we delved into exploring their in vitro antioxidant capabilities. The Ru@Mn‐ZIF nanozyme was found to display a spectrum of enzyme‐like activities under neutral conditions. Given that superoxide anion is a type of reactive oxygen species (ROS) that is overproduced in inflammatory diseases, it became imperative to assess whether the Ru@Mn‐ZIF nanozyme could exhibit superoxide dismutase (SOD)‐mimetic enzyme activity, which is crucial for scavenging oxygen‐free radicals. To this end, we utilized the xanthine oxidase method to characterize the inhibitory effect of the nanozymes on the production of oxygen‐free radicals. As depicted in Figure 2A,B, the Ru@Mn‐ZIF nanozyme demonstrated remarkable enzyme‐like activity, signifying its exceptional ability to transform free radicals. Specifically, compared with pure ZIF and Mn‐ZIF, the introduction of Ru significantly enhanced the SOD‐like enzyme activity by 19.8 and 20.8 times, respectively. This also indicates that Ru is the active center of Ru@Mn‐ZIF nanozymes. Building on these findings, we further evaluated the nanoreactor's capacity to eliminate oxygen and nitrogen free radicals using ABTS and DPPH assays. The results, as shown in Figure 2C,D, revealed that at a concentration of 200 µg/mL, the clearance rate of ABTS at 1 mg/mL could reach 80%, while the clearance rate of DPPH was approximately 20%. These findings underscore the Ru@Mn‐ZIF nanozyme's potential to effectively neutralize free radicals. In addition, we examined the changes in dissolved oxygen levels in various systems mixed with hydrogen peroxide for 10 min. The data presented in Figure 2E,F indicated that the pure ZIF, Mn‐ZIF, and Ru@Mn‐ZIF nanozyme exhibited catalase (CAT)‐like activity in phosphate buffer (pH 6.8), effectively decomposing hydrogen peroxide into oxygen. Similar to the SOD‐like enzyme activity results (Figure 2A,B), the CAT‐like activity of Ru@Mn‐ZIF is significantly superior to the enzyme activity of pure ZIF and Mn‐ZIF. We hypothesize that this discrepancy may be attributed to electron transfer between Ru and Mn, which enhances the Ru@Mn‐ZIF nanozyme's ability to scavenge oxygen and hydroxyl radicals, thereby augmenting its overall antioxidant activity. Collectively, though the activity of the Ru@Mn‐ZIF nanozymes shows significant differences from natural enzyme activity, these results highlight that the synthesized nanozyme possesses multiple enzyme activities, positioning it as a promising candidate for anti‐inflammatory and antioxidant therapies. Future work will focus on further enhancing the activity of nanozyme, elucidating the underlying mechanisms, and exploring the therapeutic potential of these nanomotors in relevant disease models.

### 2.3 Nanozyme Attenuates Lipopolysaccharide (LPS)‐Induced Oxidative Stress and Proneuroinflammation in Microglia In Vitro

**FIGURE 3:**
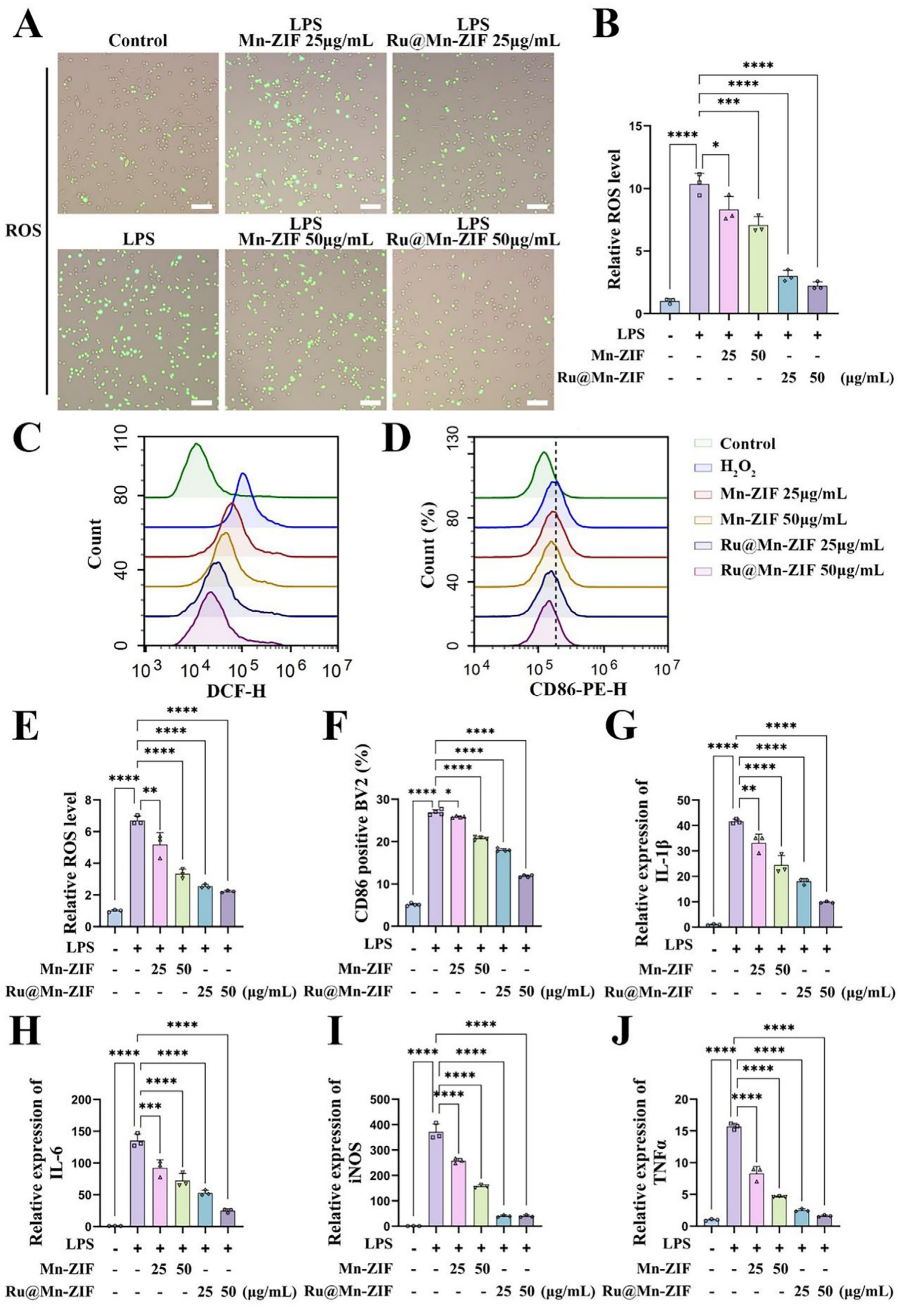
Ru@Mn‐ZIF nanozyme markedly reduces LPS‐induced oxidative stress and proinflammatory neuroimmune activation in microglia. (A, B) Fluorescence microscopy images of DCF staining in BV2 cells (Scale bars: 100 µm) and quantitative analysis of the DCF fluorescence intensity. Nanozyme pretreatment significantly attenuated LPS‐induced intracellular ROS level (*n* = 3). (C, E) Flow cytometric quantification of DCF‐labeled intracellular ROS in BV2 cells. Nanozyme pretreatment significantly attenuated LPS‐induced intracellular ROS level (*n* = 3). (D, F) Flow cytometric analysis of surface CD86 expression in BV2 cells. Nanozyme pretreatment markedly decreased LPS‐induced CD86 expression (*n* = 4). (G–J) Quantitative real‐time PCR analysis showing that nanozyme pretreatment significantly reduced LPS‐induced transcription of proinflammatory cytokines (IL‐1β, IL‐6, TNF‐α) in BV2 cells (*n* = 3). Data are presented as mean ± SEM and analyzed by one‐way ANOVA followed by Tukey's post‐hoc test. ^*^ *p* < 0.05, ^**^ *p* < 0.01, ^***^ *p* < 0.001, ^****^ *p* < 0.0001.

Microglia are the primary effector cells mediating neuroinflammation after ICH and are rapidly activated in response to injury [21, 22]. Blood components and erythrocyte degradation products that infiltrate the brain act as stimuli, driving microglia toward a proinflammatory phenotype during the acute phase of ICH and promoting excessive ROS production [23, 24]. In turn, ROS further amplifies proinflammatory activation, forming a vicious cycle of neuroinflammation and oxidative stress that exacerbates secondary brain injury [25, 26, 27, 28]. To model this condition in vitro, we stimulated BV2 microglial cells with LPS, a well‐established pro‐inflammatory agent, to induce a pro‐inflammatory and oxidative phenotype. This allowed us to evaluate the antioxidative properties of the nanozymes under neuroinflammatory conditions. First, BV2 cell viability was assessed by CCK‐8 assay after treatment with various concentrations of nanozyme for 24 h. As shown in Figure S1, both nanozymes exhibited negligible cytotoxicity within the tested range (3.125‐100 µg/mL). Based on these results and previous reports [29], concentrations of 25 and 50 µg/mL were selected for subsequent experiments. BV2 cells were pretreated with nanozyme for 6 h prior to LPS exposure to induce oxidative stress. As shown in Figure 3A–C,E, nanozyme pretreatment markedly reduced intracellular ROS levels, with Ru@Mn‐ZIF nanozyme demonstrating a stronger ROS‐scavenging effect compared with Mn‐ZIF nanozyme. We next examined the expression of the proinflammatory surface marker CD86 of microglia by flow cytometry. As shown in Figure 3D,F, LPS stimulation significantly increased CD86 expression on BV2 cells, whereas nanozyme pretreatment for 6 h markedly reduced CD86 expression. Notably, Ru@Mn‐ZIF nanozyme produced a greater reduction in CD86 levels compared with Mn‐ZIF nanozyme, in a concentration‐dependent manner. To further assess the ability of Ru@Mn‐ZIF nanozyme to inhibit microglial proinflammatory activation, quantitative real‐time PCR was performed to measure the mRNA expression of IL‐1β, IL‐6, and TNF‐α. As shown in Figure 3G–J, LPS stimulation robustly upregulated the transcription of these proinflammatory cytokines, whereas nanozyme pretreatment significantly suppressed their expression. Consistent with the ROS scavaging and anti‐proneuroinflammation findings, Ru@Mn‐ZIF nanozyme produced a greater inhibitory effect than Mn‐ZIF nanozyme in a concentration‐dependent manner.

### 2.4 Nanozyme Attenuates Hydrogen Peroxide (H_2_O_2_)‐Induces Oxidative Stress and Neuronal Death in Mouse Neurons In Vitro

**FIGURE 4:**
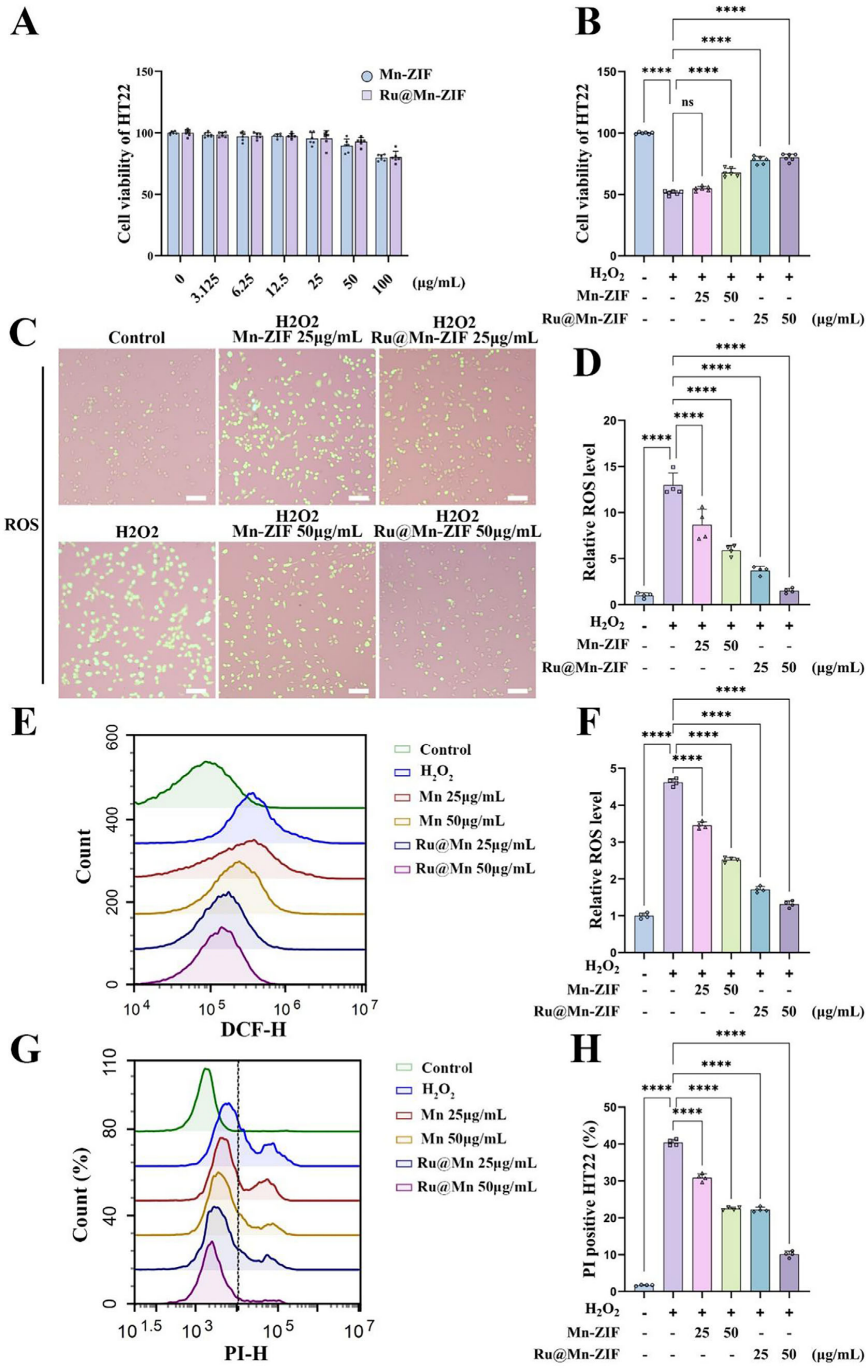
Ru@Mn‐ZIF nanozyme significantly reduces H_2_O_2_‐induced oxidative stress and neuronal death in HT22 cells. (A) CCK‐8 assay of HT22 cell viability after 24 h treatment with different concentrations of nanozyme (*n* = 6). (B) Cell viability of HT22 neurons pretreated with nanozyme for 6 h followed by 2 h exposure to H_2_O_2_. Ru@Mn‐ZIF nanozyme markedly improved neuronal survival compared with Mn‐ZIF nanozyme (*n* = 6). (C, D) Fluorescence microscopy images of DCF staining in HT22 cells (Scale bars: 100 µm) and quantitative analysis of the DCF fluorescence intensity. Nanozyme pretreatment significantly attenuated H_2_O_2_‐induced intracellular ROS level (*n* = 4). (E, F) Flow cytometric quantification of DCF‐labeled intracellular ROS in HT22 cells. Nanozyme pretreatment significantly decreased H_2_O_2_‐induced ROS accumulation (*n* = 4). (G, H) Flow cytometric analysis of PI staining showing that nanozyme pretreatment significantly reduced H_2_O_2_‐induced neuronal death (*n* = 4). Data are presented as mean ± SEM and analyzed by one‐way ANOVA followed by Tukey's post‐hoc test. ^*^ *p* < 0.05, ^**^ *p* < 0.01, ^***^ *p* < 0.001, ^****^ *p* < 0.0001.

At the onset of ICH, neurons are subjected to irreversible damage due to the sudden mass effect within the brain parenchyma. Surviving neurons are further exposed to oxidative stress and proinflammatory insults in the perihematomal microenvironment [30, 31]. To assess the direct neuroprotective and antioxidative effects of the nanozymes on neurons under oxidative insult, we exposed HT22 hippocampal neuronal cells to H_2_O_2_, a representative reactive oxygen species ROS, to establish an oxidative injury model. As shown in Figure 4A, CCK‐8 assays revealed that treatment of HT22 cells with Mn‐ZIF or Ru@Mn‐ZIF nanozyme for 24 h maintained cell viability above 80% at concentrations up to 100 µg/mL, although a decline was observed at this highest concentration. Based on these findings and previous reports [29], concentrations of 25 and 50 µg/mL were selected for subsequent experiments. Pretreatment of HT22 cells with nanozyme for 6 h significantly increased cell viability after 24 h exposure to H_2_O_2_ (Figure 4B). To directly assess the effect of nanozyme on intracellular ROS levels under oxidative stress, DCF‐labeled ROS were quantified by fluorescence microscope and flow cytometry. As shown in Figure 4C–F, both Mn‐ZIF and Ru@Mn‐ZIF nanozymes markedly reduced intracellular ROS levels in H_2_O_2_‐exposed HT22 cells, with Ru@Mn‐ZIF nanozyme demonstrating superior ROS scavenging ability. Furthermore, propidium iodide (PI) staining combined with flow cytometry was used to evaluate neuronal death under oxidative stress conditions. As shown in Figure 4G,H, nanozyme pretreatment significantly reduced H_2_O_2_‐induced neuronal death in a concentration‐dependent manner. Flow cytometry analysis revealed a bimodal distribution of PI staining in H_2_O_2_‐stimulated cells, corresponding to distinct populations of early and late apoptotic neurons, an effect that was mitigated by nanozyme treatment (Figure 4G). Consistent with the ROS clearance results, Ru@Mn‐ZIF nanozyme exerted stronger neuroprotective effects compared with Mn‐ZIF nanozyme.

### 2.5 Different Administration Routes of Nanozyme Attenuate Hematoma Volume and BBB Leakage and Improve Neurological Function in Collagenase‐Induced ICH Mice

To evaluate the therapeutic efficacy of nanozyme delivered via different administration routes, a collagenase‐induced ICH mouse model was established, and nanozyme were administered either intranasally or intravenously 1 h after model induction. Neurological performance and histopathological changes were comprehensively assessed.

**FIGURE 5:**
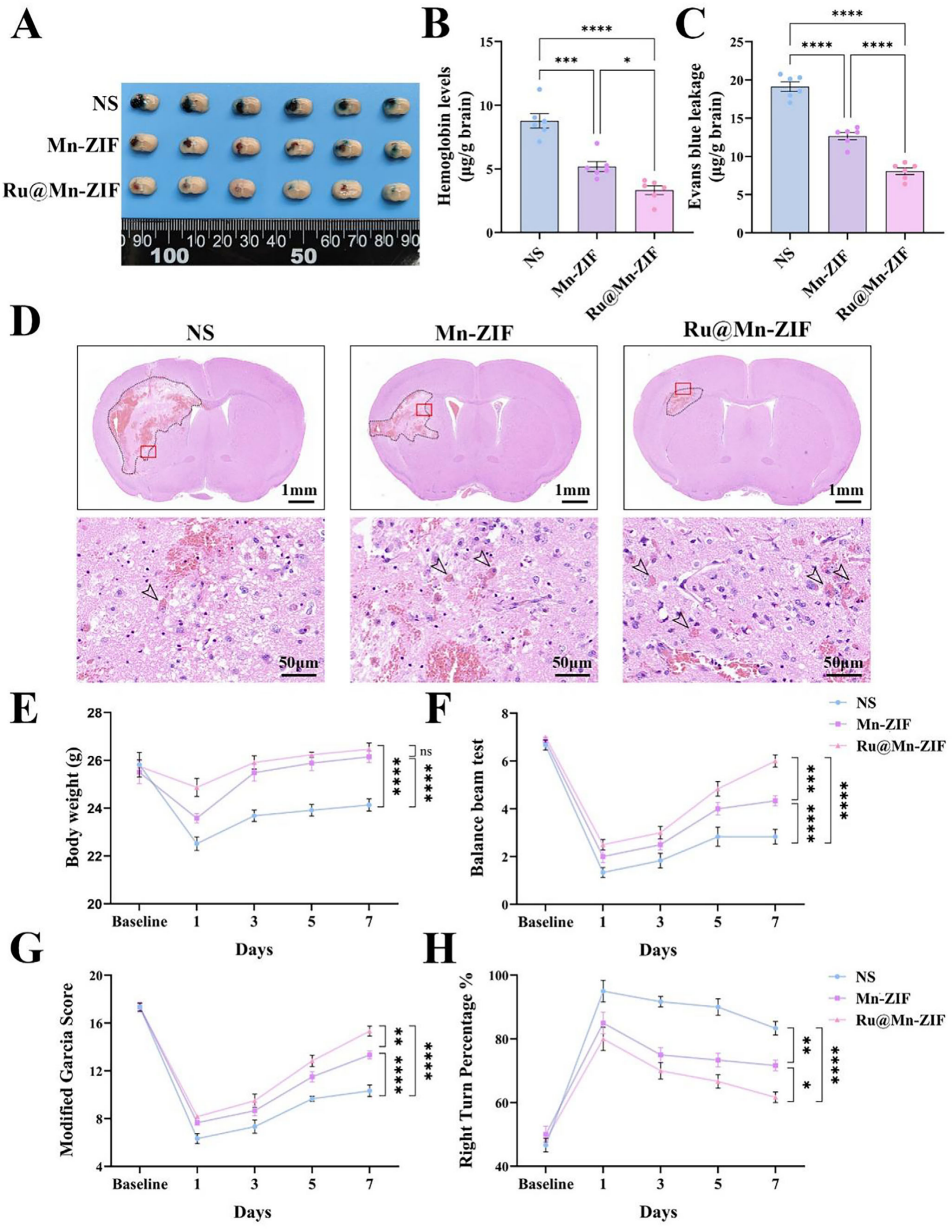
Therapeutic evaluation of intranasally administered nanozyme in collagenase‐induced ICH mice. (A–C) Intranasal nanozyme administration significantly reduced hematoma volume and Evans blue extravasation in the brain 3 days post‐treatment (*n* = 6). Data are presented as mean ± SEM and analyzed using one‐way ANOVA followed by Tukey's post‐hoc test. ^*^ *p* < 0.05, ^***^ *p* < 0.001, ^****^ *p* < 0.0001. (D) H&E staining demonstrated a reduction in perihematomal edema and inflammatory cell infiltration on day 3 following intranasal nanozyme treatment. In the Ru@Mn‐ ZIF group, numerous phagocytes containing engulfed red blood cells were observed (arrows). Upper panel, 2× magnification; lower panel, 40× magnification. Scale bars: 1 mm (upper), 50 µm (lower). (E) Intranasal nanozyme administration significantly reversed body weight loss in ICH mice (*n* = 6). (F, G) Intranasal nanozyme treatment markedly improved neurological performance, as indicated by increased balance beam test scores and modified Garcia scores (*n* = 6). (H) Intranasal Ru@Mn nanozyme administration significantly reduced the percentage of right turns in the corner turn test (*n* = 6). Data are presented as mean ± SEM and analyzed using two‐way ANOVA followed by Tukey's post‐hoc test. ^*^ *p* < 0.05, ^**^ *p* < 0.01, ^***^ *p* < 0.001, ^****^ *p* < 0.0001.

Intranasal delivery is a noninvasive route that allows drugs to bypass the BBB through the olfactory mucosa and directly enter the brain [32, 33]. As shown in Figure 5A–C, intranasal administration of nanozyme significantly reduced brain hemoglobin content and Evans blue extravasation in the ipsilateral hemisphere on day 3 after ICH. Compared with Mn‐ZIF nanozyme, Ru@Mn‐ZIF nanozyme further reduced hematoma volume and BBB leakage. Consistently, HE staining (Figure 5D) demonstrated decreased inflammatory cell infiltration in perihematomal regions, accompanied by numerous phagocytes engulfing erythrocytes after nanozyme treatment. Intranasal nanozyme administration also significantly reversed ICH‐induced body weight loss (Figure 5E). As shown in Figure 5F–H, neurological outcomes gradually improved over time, with higher modified Garcia scores and balance beam scores and reduced turning bias. Compared with the NS group, nanozyme treatment markedly improved neurological function, with Ru@Mn‐ZIF nanozyme exhibiting superior efficacy over Mn‐ZIF nanozyme.

To further evaluate histopathological changes, multiple staining methods were performed on perihematomal brain tissue on day 7 after ICH. As shown in Figure S2A, nanozyme treatment significantly reduced glial scar formation. Perls’ Prussian blue staining confirmed markedly decreased iron deposition (Figure S2B, C), while Luxol fast blue staining revealed attenuated myelin loss in perihematomal white matter (Figure S2D,E). Notably, Ru@Mn‐ZIF nanozyme conferred greater neuroprotection compared with Mn‐ZIF nanozyme.

Intravenous injection, a clinically common administration route, was also assessed in ICH mice. As shown in Figure S3A–C, intravenous nanozyme administration significantly reduced hematoma volume and BBB disruption on day 3 after ICH, and alleviated pathological features such as perihematomal inflammation and cerebral edema (Figure S3D). Analysis of body weight and neurological function (Figure S3E‐H) indicated that intravenous Mn‐ZIF nanozyme provided less functional improvement than intranasal delivery, whereas intravenous Ru@Mn‐ZIF nanozyme exhibited robust efficacy in ameliorating neurological deficits. Histopathological evaluation on day 7 (Figure S4) further demonstrated that intravenous nanozyme treatment mitigated glial scar formation, iron deposition, and myelin loss in ICH mice.

Collectively, these findings suggest that Ru@Mn‐ZIF nanozyme outperforms Mn‐ZIF nanozyme in alleviating hematoma burden, preserving BBB integrity, and improving neurological outcomes, regardless of the administration route, thereby supporting their potential for future translational applications.

### 2.6 Nanozyme Treatment Reduces Inflammatory Cell Infiltration and Activation and Suppresses Proinflammatory Cytokine Release in ICH Mice

**FIGURE 6:**
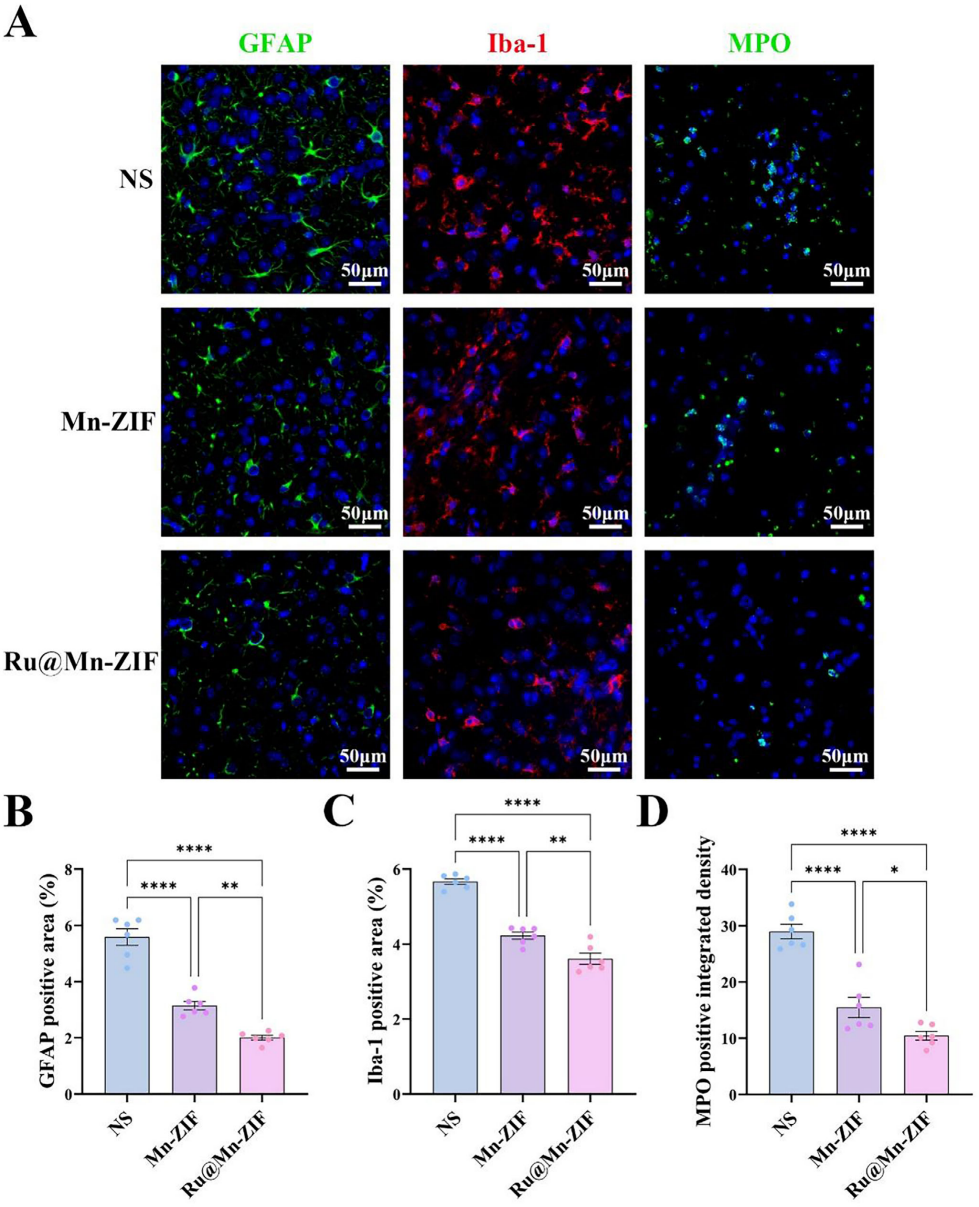
Inflammatory cell activation in the perihematomal region of ICH mice on day 3 following intranasal nanozyme treatment. (A) Immunofluorescence staining of major inflammatory cells in perihematomal brain tissue: astrocytes (GFAP), microglia/macrophages (Iba‐1), and neutrophils (MPO). Scale bars: 50 µm. (B) Quantification of GFAP‐positive astrocytic area (*n* = 6). (C) Quantification of Iba‐1–positive microglial/macrophage area (*n* = 6). (D) Quantification of MPO‐positive neutrophil fluorescence intensity (*n* = 6). Data are presented as mean ± SEM and analyzed using one‐way ANOVA followed by Tukey's post‐hoc test. ^*^ *p* < 0.05, ^**^ *p* < 0.01, ^****^ *p* < 0.0001.

To further evaluate the therapeutic effects of nanozyme treatment on neuroinflammation in ICH, immunofluorescence and immunohistochemistry were performed to detect inflammatory cell infiltration and activation in perihematomal brain tissue on day 3 post‐ICH. As shown in Figure 6A, robust activation of astrocytes (GFAP) and microglia (Iba‐1) was observed in the NS group, accompanied by strong MPO‐positive fluorescence signals indicating neutrophil infiltration in both perihematomal and intralesional regions. Quantitative analysis revealed that intranasal nanozyme treatment significantly reduced astrocytic and microglial activation, as well as neutrophil infiltration (Figure 6B–D). Similar therapeutic effects were also observed in ICH mice treated with intravenous nanozyme administration (Figure S5).

**FIGURE 7:**
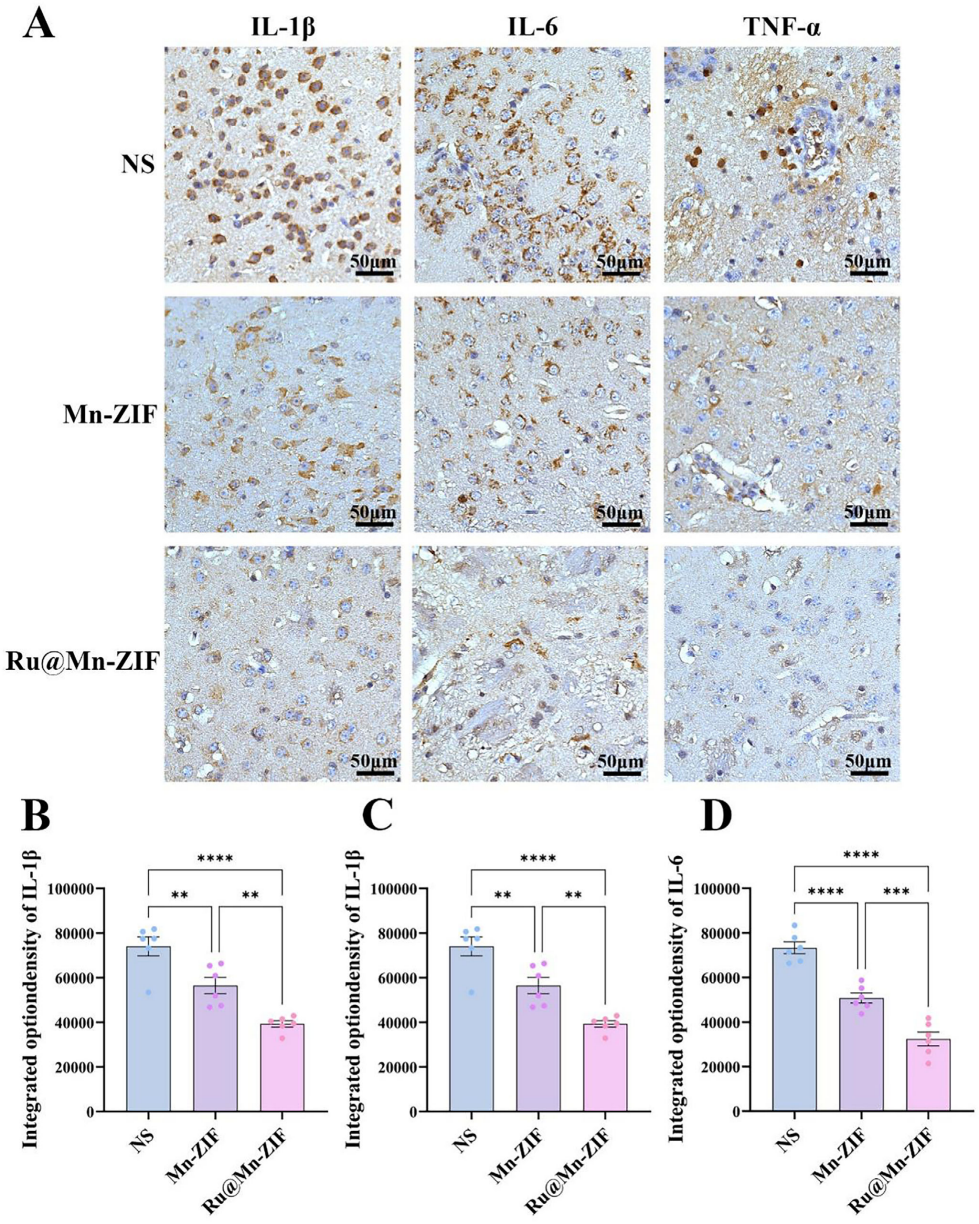
Expression of proinflammatory cytokines in the perihematomal region of ICH mice on day 3 following intranasal nanozyme treatment. (A) Immunohistochemical staining of major proinflammatory cytokines in perihematomal brain tissue. Scale bars: 50 µm. (B) Quantification of IL‐1β immunohistochemical DAB integrated optical density (IOD) (*n* = 6). (C) Quantification of IL‐6 immunohistochemical DAB IOD (*n* = 6). (D) Quantification of TNF‐α immunohistochemical DAB IOD (*n* = 6). Data are presented as mean ± SEM and analyzed using one‐way ANOVA followed by Tukey's post‐hoc test. ^**^ *p* < 0.01, ^***^ *p* < 0.001, ^****^ *p* < 0.0001.

During the acute phase of ICH, activated inflammatory cells release abundant proinflammatory cytokines such as IL‐1β, IL‐6, and TNF‐α, which further aggravate brain injury [34]. To assess cytokine release, immunohistochemistry was performed on perihematomal tissue collected on day 3 post‐ICH. As shown in Figure 7A, large amounts of proinflammatory cytokines were detected in the NS group, whereas intranasal nanozyme treatment markedly reduced cytokine expression. Consistent results were observed in the intravenous administration group (Figure S6). Importantly, Ru@Mn‐ZIF nanozyme demonstrated greater efficacy than Mn‐ZIF nanozyme in reducing inflammatory cell infiltration and activation as well as suppressing proinflammatory cytokine release, regardless of the administration route.

### 2.7 Nanozyme Treatment Increases the Number of Surviving Neurons and Attenuates Oxidative Stress‐Induced Neuronal Damage in ICH Mice

**FIGURE 8:**
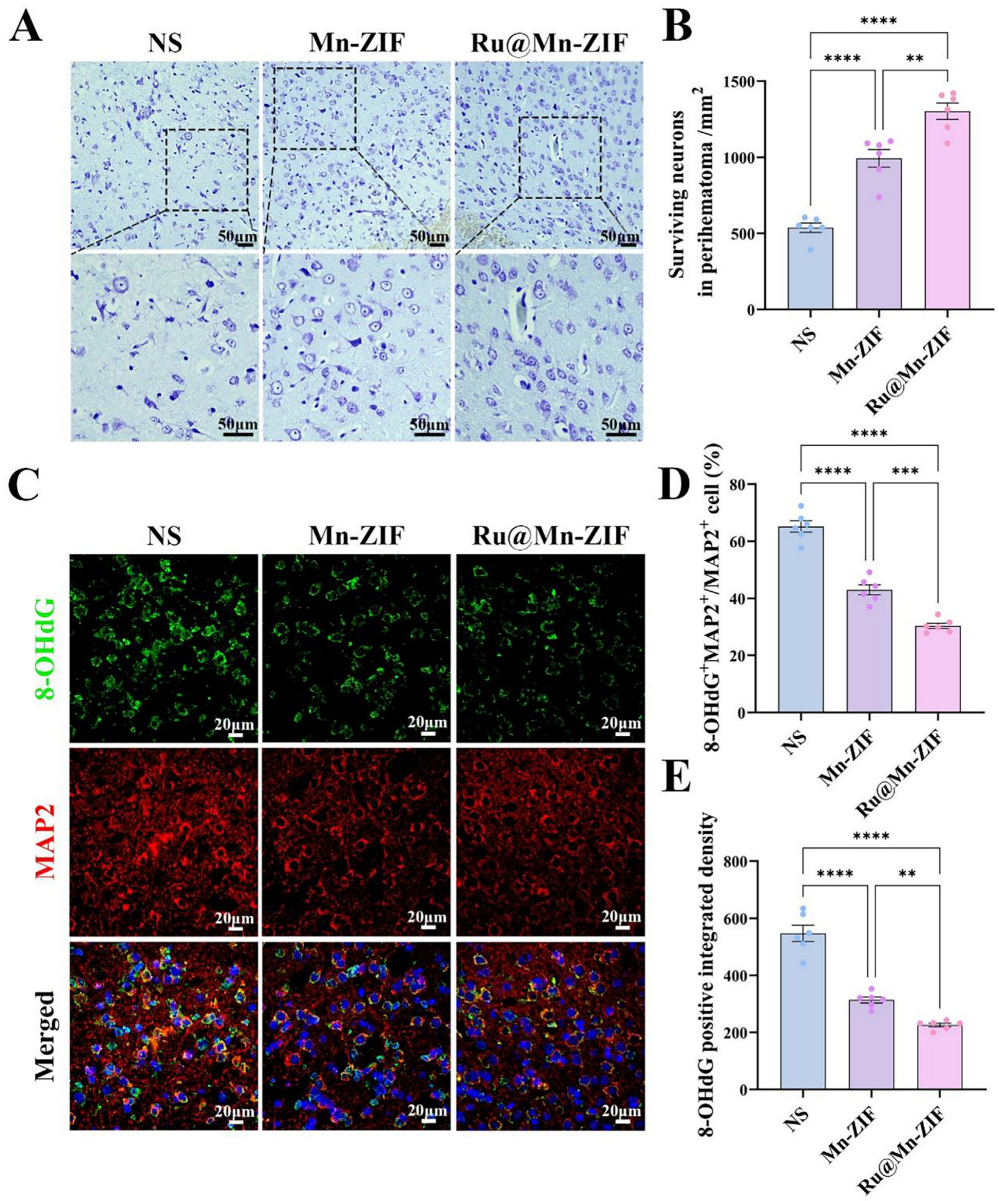
Evaluation of perihematomal neuronal survival and DNA oxidative stress injury in collagenase‐induced ICH mice after 3 days of intranasal nanozyme treatment. (A) Nissl staining of perihematomal brain tissue (upper panel, 20× magnification; lower panel, 40× magnification). Scale bars: 20 µm. (B) Quantification of perihematomal surviving neuronal density based on Nissl staining (*n* = 6). (C) Immunofluorescence staining of neuronal DNA oxidative stress markers in perihematomal regions: 8‐OHdG (green) and MAP2 (red). Scale bars: 20 µm. (D) Proportion of 8‐OHdG–positive neurons among total neurons in perihematomal regions (*n* = 6). (E) Quantification of 8‐OHdG–positive fluorescence intensity in perihematomal regions (*n* = 6). Data are presented as mean ± SEM and analyzed by one‐way ANOVA followed by Tukey's post‐hoc test. ^**^ *p* < 0.01, ^***^ *p* < 0.001, ^****^ *p* < 0.0001.

The basal ganglia are the most common site of primary ICH, and given their role as a critical relay station in somatosensory and motor pathways, neuronal survival in this region forms the structural basis for functional recovery after ICH [35]. To assess the neuroprotective effects of nanozyme treatment on striatal neurons, Nissl staining was performed to evaluate perihematomal neuronal density. As shown in Figure 8A,B, intranasal administration of Ru@Mn‐ZIF nanozyme markedly increased neuronal density compared with the other groups. Correlation analysis further demonstrated that perihematomal neuronal density was significantly positively correlated with beam balance scores and modified Garcia scores, while negatively correlated with the percentage of right turns (Figure S7). These findings provide evidence explaining the significant improvement in neurological function observed with Ru@Mn‐ZIF nanozyme treatment. Consistent results were also observed in ICH mice treated via intravenous administration (Figure S8). In the meantime, excessive activation of inflammatory cells following ICH generates high levels of ROS, leading to severe oxidative stress–induced neuronal injury. To investigate whether nanozyme treatment protects neurons from oxidative stress in perihematomal regions, double immunofluorescence staining was used to assess the proportion of neurons with oxidative DNA damage. As shown in Figure 8C–E, abundant 8‐OHdG‐positive neurons were observed in perihematomal regions of untreated NS mice. In contrast, nanozyme treatment significantly reduced the proportion of 8‐OHdG‐positive neurons, with Ru@Mn‐ZIF nanozyme producing a more pronounced protective effect than Mn‐ZIF nanozyme. Similarly, intravenous nanozyme administration also reduced oxidative stress–induced neuronal damage (Figure S9), although the effect was less robust compared with intranasal administration.

### 2.8 Intranasal Nanozyme Administration Improves Neurological Deficits and Histopathological Damage in Autologous Blood‐Induced ICH Mice

To further evaluate the therapeutic efficacy of nanozyme across different ICH induction methods, an autologous blood injection model was established by intrastriatal infusion of autologous arterial blood. Based on the above results showing superior efficacy via the intranasal route, we next assessed the therapeutic potential of intranasal nanozyme administration in an autologous blood–induced ICH mice model. As shown in Figures S10A–C, intranasal nanozyme treatment significantly reduced hematoma volume and BBB leakage in autologous blood–induced ICH mice, with Ru@Mn‐ZIF nanozyme producing greater therapeutic benefit than Mn‐ZIF nanozyme. Consistently, HE staining (Figure S10D) revealed increased numbers of phagocytes engulfing erythrocytes in perihematomal regions after nanozyme treatment. Evaluation of body weight and neurological function further demonstrated that Ru@Mn‐ZIF nanozyme provided superior improvement in reversing weight loss and ameliorating neurological deficits compared with Mn‐ZIF nanozyme. Moreover, Nissl staining revealed that intranasal nanozyme administration markedly increased the number of surviving neurons in perihematomal regions, and correlation analysis confirmed a significant positive association between neuronal survival and improved neurological performance (Figure S11). Together, these findings confirm that intranasal Ru@Mn‐ZIF nanozyme administration effectively alleviates hematoma burden, preserves BBB integrity, and improves neurological recovery in the autologous blood–induced ICH model.

### 2.9 Biosafety Evaluation of Nanozyme Treatment in ICH Mice

To assess the biosafety of nanozyme treatment in ICH mice, peripheral blood and major organs were collected on day 7 after administration for serum biochemical analysis and histopathological examination. As shown in Figures S12 and S13, HE staining of major organs revealed no abnormal pathological changes in either the intranasal or intravenous nanozyme treatment groups compared with the NS group. Similarly, serum biochemical parameters from peripheral blood (Figures S14 and S15) showed no significant alterations among the three groups, and all values remained within the normal reference range. These results indicate that nanozyme treatment is well‐tolerated and exhibits a favorable biosafety profile via both intranasal and intravenous administration.

## 3 Conclusion

In summary, we developed Ru‐Mn composite nanozyme with cascade catalytic activity to efficiently scavenge ROS and modulate neuroinflammation in ICH. Both intranasal and intravenous administration significantly reduced hematoma volume, preserved BBB integrity, attenuated inflammatory responses, and improved neuronal survival and neurological recovery in collagenase and autologous blood–induced ICH models. Importantly, biosafety assessments confirmed favorable tolerability without pathological abnormalities or biochemical alterations. These findings highlight Ru@Mn‐ZIF nanozyme as a promising therapeutic strategy for mitigating secondary brain injury and improving outcomes after ICH, offering strong potential for clinical translation.

## 4 Experimental Section

### 4.1 Synthesis of Ru@Mn‐ZIF

Mn‐ZIF was first synthesized by dropwise addition of Solution A (0.1 mmol dopamine, 0.125 mmol Zn(NO_3_)_2_·6H_2_O, and 0.125 mmol Mn(NO_3_)_2_·4H_2_O in 1 mL H_2_O) into Solution B (1.27 g 2‐methylimidazole in 9 mL H_2_O) under stirring. After 12 h reaction at room temperature, the precipitate was washed and freeze‐dried to obtain Mn‐ZIF. To prepare Ru@Mn‐ZIF, Mn‐ZIF (20 mg) and RuCl_3_·3H_2_O (2 mg) were dispersed in 10 mL H_2_O and stirred for 12 h. Subsequently, 1 mL of NaBH_4_ solution (2 mg/mL) was added dropwise. After 5 min, the product was collected by centrifugation and lyophilized.

### 4.2 SOD‐Like Activity of Ru@Mn‐ZIF

SOD‐like activity was assessed via the cytochrome C reduction method using the xanthine/xanthine oxidase system. The O_2_•^−^ generation rate was monitored by the absorbance increase of cytochrome C at 550 nm. Inhibition rates at different nanozyme concentrations were calculated as (ΔA_1_ – ΔA_2_)/ΔA_1_ × 100%, where ΔA_1_ and ΔA_2_ represent the absorbance changes of the control and test groups, respectively.

### 4.3 CAT‐Like Activity of Ru@Mn‐ZIF

CAT‐like activity was evaluated by monitoring O_2_ production in 0.3% H_2_O_2_ solution using a dissolved oxygen meter, and by tracking H_2_O_2_ decomposition via absorbance decrease at 240 nm. The reaction mixture contained 40 µg nanozyme in PBS buffer with 0.3% H_2_O_2_.

### 4.4 Radical Scavenging Assays (ABTS and DPPH)

ABTS•^+^ radicals were generated by mixing 7 mM ABTS with 2.45 mm potassium persulfate for 16 h. After adding Ru@Mn‐ZIF (0–1 mg/mL), absorbance at 734 nm was measured. DPPH scavenging was assessed by mixing nanozyme with 125 µm DPPH in ethanol (1:1 v/v). After 30 min, absorbance at 517 nm was recorded. Scavenging activity was calculated as [1 – (Ai—A_1_)]/A_0_ × 100%.

### 4.5 Cell Culture and Oxidative Stress Models

BV2 microglia and HT22 hippocampal neurons (Procell, China) were cultured in DMEM containing 10% FBS and 1% penicillin/streptomycin. To establish oxidative stress models, BV2 cells were stimulated with LPS (1 µg/mL, 24 h), and HT22 cells with H_2_O_2_ (0.1 mm, 24 h). For treatment, cells were pretreated with nanozymes (25 or 50 µg/mL) for 6 h prior to stimulation.

### 4.6 Cell Viability Assay

Cells seeded in 96‐well plates (6,000 cells/well) were treated with nanozymes (3.125–100 µg/mL) for 24 h. Viability was measured using the CCK‐8 assay (10 µL/well, 1 h incubation at 37°C) by absorbance at 450 nm.

### 4.7 Intracellular ROS Detection

After pretreatment and stimulation, cells were incubated with 10 µm DCFH‐DA at 37°C for 30 min. Fluorescence intensity was analyzed by flow cytometry (NovoCyte, Agilent) and fluorescence microscopy (Zeiss).

### 4.8 Anti‐Neuroinflammation Assay In Vitro

BV2 cells were pretreated with nanozymes (25 or 50 µg/mL, 6 h) and stimulated with LPS (1 µg/mL, 24 h). CD86 surface expression was analyzed by flow cytometry after PE‐conjugated antibody staining. mRNA levels of IL‐1β, IL‐6, iNOS, and TNF‐α were quantified by qPCR (primer sequences in Table S1).

### 4.9 Neuronal Oxidative Stress Injury Assay In Vitro

HT22 cells were pretreated with nanozymes (25 or 50 µg/mL, 6 h) and exposed to H_2_O_2_ (0.1 mm, 2 h). Cell death was assessed by propidium iodide (PI) staining followed by flow cytometry (BD Pharmingen, No. 556463).

### 4.10 Animals and Ethical Approval

Male C57BL/6J mice (6–8 weeks) were obtained from Beijing Huafukang Biotechnology Co., Ltd. All procedures were approved by the Animal Ethics Committee of the State Key Laboratory of Biotherapy, Sichuan University (Approval No. 20250314030) and complied with the Guide for the Use of Laboratory Animals.

### 4.11 ICH Mouse Model and Treatment

ICH was induced by stereotaxic injection of collagenase VII (0.5 µL at 0.1 µL/min) or autologous arterial blood (30 µL at 3 µL/min) into the right striatum (coordinates: 0.5 mm anterior, 2.0 mm lateral, 3.5 mm depth). One hour post‐ICH, mice received nanozymes (8 mg/kg) via intranasal instillation (20 µL total) or intravenous injection (100 µL) once daily for 3 days. Neurological function was assessed on days 1, 3, 5, and 7 using beam balance, modified Garcia score, and circling tests. Body weight was recorded throughout.

### 4.12 Histopathological Assessment of Brain Tissue

On day 3 post‐ICH, BBB integrity was evaluated by Evans blue leakage, and hemoglobin content was quantified using Drabkin's reagent. Brain sections were stained with hematoxylin‐eosin (HE), Nissl, and Perls' Prussian blue. Immunohistochemistry for IL‐1β, IL‐6, and TNF‐α, and immunofluorescence for 8‐OHdG, Iba‐1, GFAP, and MPO were performed on perihematomal tissues.

### 4.13 Biosafety Assessment of Nanozymes Treatment

On day 7, blood was collected for serum biochemistry. Major organs (heart, liver, spleen, lungs, kidneys) were harvested and processed for HE staining.

### 4.14 Statistical Analysis

All data are expressed as mean ± SEM. Statistical analyses were conducted using GraphPad Prism 10.0. Group comparisons were performed using one‐way ANOVA, two‐way ANOVA, or Student's *t*‐test, and correlations were evaluated by Spearman's correlation coefficient. A p‐value < 0.05 was considered statistically significant (^*^ *p* < 0.05, ^**^ *p* < 0.01, ^***^ *p* < 0.001, ^****^ *p* < 0.0001).

Further detailed experimental procedures are available in the Supporting Information.

## Conflicts of Interest

The authors declare no conflict of interest.

## Supporting information




**Supporting File**: advs74804‐sup‐0001‐SuppMat.docx

## Data Availability

Research data are not shared.
