# Supplementary material for: Biomimetic Ru‐Mn Nanozyme with Cascade Catalytic Activity Attenuates Secondary Brain Injury in Intracerebral Hemorrhage
Source: Adv Sci (Weinh). 2026 Mar 13;13(41):e19340. doi: 10.1002/advs.202519340 (PMC13325807; doi:10.1002/advs.202519340)
Supplement: Supplementary file 1 — Supporting File: advs74804‐sup‐0001‐SuppMat.docx [file ADVS-13-e19340-s001.docx]

**Supplementary Information**

**Experimental Section**

***Materials***

2-Methylimidazole, 2,2’-azinobis-(3-ethylbenzthiazoline-6-sulfonic acid) (ABTS), 3,3’,5,5’-tetramethylbenzidine (TMB), (GOx) was purchased from Macklin reagent Co., Ltd. (Shang-hai, China). Mn(NO_3_)_2_·4H_2_O_,_ Zn(NO_3_)_2·_6H_2_O, and RuCl_3_·3H_2_O were purchased from Sinopharm Chemical Reagent Co. Ltd. All solutions prepared are derived from Wahaha purified water (China). All other chemicals were of the highest grade and used with-out further purification unless otherwise noted.

***Instrumentation***

Scanning electron microscopy (SEM) was conducted using Hitachi FE-SEM S-4800 at 3 kV. X-ray photoelectron spectroscopy (XPS) spectra were measured using Thermo ESCALAB 250Xi. The dissolved oxygen meter was operated with Mettler Toledo M400. UV-vis spectra were recorded on the UV-2700 spectrophotometer manufactured by Shimadzu (Japan).

***Synthesis of Ru@Mn-ZIF***

In this experimental procedure, a solution designated as Solution A is prepared by dissolving 0.1 mmol of dopamine, 0.125 mmol of Zn(NO_3_)_2_·6H_2_O, and 0.125 mmol of Mn(NO_3_)_2_·4H_2_O in 1 mL of deionized water. For Solution B, 1.27 g of 2-Methylimidazole is dissolved in 9 mL of deionized water. After thorough stirring of Solution B, Solution A is added dropwise to Solution B. The mixture is then allowed to react for 12 hours at room temperature.

Following the reaction period, the resulting precipitate is washed with deionized water three to five times until the supernatant is clear. The washed precipitate is then subjected to freeze-drying to obtain the Mn-ZIF product. The morphology of the Mn-ZIF was optimized by adjusting the molar ratios of Zn(NO_3_)_2_·6H_2_O to Mn(NO_3_)_2_·4H_2_O, with specific ratios of 1:4, 2:3, 3:2, and 4:1, while maintaining a total molar amount of 0.25 mmol.

For the subsequent step, RuCl_3_·3H_2_O (2 mg) and Mn-ZIF (20 mg) are added to 10 mL of deionized water and stirred for 12 hours. Subsequently, NaBH_4_ aqueous solution (2 mg/mL, 1 mL) is added dropwise to the mixture under vigorous stirring. The reaction is allowed to proceed at room temperature for 5 minutes. The final Ru@Mn-ZIF product is collected by centrifugation and then lyophilized for further use.

This refined procedure ensures precise control over the synthesis conditions, leading to the optimized morphology and functionalization of the Ru@Mn-ZIF nanomaterial.

***SOD-like activity of Ru@Mn-ZIF***

The cytochrome C reduction technique was used to estimate the O_2_^•-^ generated by the xanthine-Xanthine oxidase system. A certain quantity of oxidised cytochrome C was reduced to reduced cytochrome C, which showed maximum light absorption at 550 nm. Because SOD disproportionately lowered the response speed of O_2_^•-^ reducing cytochrome C in the presence of SOD.

2O_2_^•-^+ 2H^+^ = H_2_O_2_+O_2_

The O_2_^•-^ concentration of the control system (300 µl in total: xanthine 50 µl, cytochrome C 50 µl, xanthine oxidase and ddH2O 150 µl, PBS buffer: 50 µl) was detected by measuring the increase in the absorbance at 550 nm (ΔA1) of cytochrome C by Multifunctional microplate reader in 1 minute. The absorbance of minute 0 is denoted as ΔA1, the absorbance of minute 1 is denoted as ΔA2, and the difference between ΔA2 and ΔA1 is 0.0225±0.001 by adjusting the amount of xanthine oxidase and deionized water, then the amount of xanthine oxidase at this time is the subsequent dosage. The inhibition rate calculated by the absorbance obtained by setting different concentration gradients in the nanozyme detection system (300 µl in total: xanthine 50 µL; cytochrome C 50 µl; xanthine oxidase, nanozyme, and ddH_2_O 150 µl; buffer: 50 µL) can be used as the inhibition rate curve. The O_2_^•−^ elimination rate of nanozymes was calculated as (ΔA1-ΔA2)/ΔA1*100%.

***CAT-like activity of Ru@Mn-ZIF***

The CAT-like activity of nanozymes was measured by monitoring the increase in O_2_ concentration in 0.3% H_2_O_2_ solution using a Dissolved Oxygen Meter (InPro 6860i, Mettler Toledo, Switzerland). The reaction system contains 40 µg nanozyme and 0.3% H_2_O_2_ in PBS buffer. The decomposition of H_2_O_2_ by nanozymes was measured by monitoring the decrease in the absorbance at 240 nm of H_2_O_2_.

***ABTS radical-scavenging activity of Ru@Mn-ZIF***

The ABTS radical cation discoloration test was utilized to measure the free radical scavenging capacity of the prepared nanozymes. First, ABTS radical cations (ABTS^+^) were generated by mixing 7 mM ABTS aqueous solution with 2.45 mM potassium persulfate and incubating for 16 hours. Next, different concentrations of Ru@Mn-ZIF (0-1 mg/mL) were added to the above mixture. Finally, the absorbance at 734 nm of the resulting solutions was measured using a spectrophotometer, and the scavenging efficiency of ABTS was calculated as follows:

ABTS radical cations scavenging activity=[1－(A_i_－A_1_)]/A_0_$\times$100%.

A_0_ is the absorbance of ABTS solutions without adding samples; A_i_ is the absorbance of the sample after reacting with ABTS; A_1_ is the absorbance of solvent mixed with nanozymes.

***DPPH radical cations scavenging activity of Ru@Mn-ZIF***

Different concentrations of Ru@Mn-ZIF were prepared and mixed with DPPH (125 μM) in equal volumes of ethanol. The final concentrations of Ru@Mn-ZIF ranged from 0 to 1 mg/mL, while the final DPPH concentration was 62.5 μM. After a 30 min reaction period, the absorbance of DPPH at 517 nm was measured. The scavenging efficiency of DPPH was calculated using the following equation:

DPPH radical cations scavenging activity=[1－(A_i_－A_1_)]/A_0_×100%.

A_0_ is the absorbance of DPPH solutions without adding samples; A_i_ is the absorbance of the sample after reacting with DPPH solutions; A_1_ is the absorbance of solvent mixed with nanozymes.

***Establishment of oxidative stress injury models in vitro and treatment***

Mouse microglial BV2 cells and hippocampal neuronal HT22 cells were obtained from Procell Biotechnology (Wuhan, China). BV2 cells were cultured in DMEM supplemented with 10% heat-inactivated fetal bovine serum (FBS, 56 °C, 30 min) and 1% penicillin/streptomycin, while HT22 cells were maintained in DMEM with 10% FBS and 1% penicillin/streptomycin. To evaluate the therapeutic potential of the nanozymes, cells were pretreated with different concentrations of nanozymes for 6 h, followed by stimulation with LPS (1 μg/mL, 24 h) for BV2 cells or H₂O₂ (0.1 mM, 24 h) for HT22 cells to establish in vitro oxidative stress injury models.

***Cell viability assay***

BV2 or HT22 cells were seeded into 96-well plates at 6,000 cells/well and cultured overnight. Cells were then treated with increasing concentrations of nanozymes (3.125–100 μg/mL) for 24 h. Cell viability was assessed using the CCK-8 assay according to the manufacturer’s protocol by adding 10 μL of CCK-8 reagent to each well containing 100 μL medium, followed by incubation at 37 °C for 1 h. Absorbance at 450 nm was measured using a microplate reader (Thermo Fisher).

To assess the protective effect of the nanozymes against oxidative stress, HT22 cells were pretreated with 25 or 50 μg/mL nanozymes for 6 h, followed by exposure to H₂O₂ (0.1 mM, 24 h). Cell viability was subsequently measured using the CCK-8 assay as described above.

***Intracellular ROS detection***

BV2 and HT22 cells were seeded into 6-well plates at 1.5 × 10⁴ cells/mL and cultured overnight. Cells were pretreated with 25 or 50 μg/mL nanozymes for 6 h, followed by stimulation with LPS (1 μg/mL, 24 h) for BV2 cells or H₂O₂ (0.1 mM, 2 h) for HT22 cells. Intracellular ROS levels were detected using 10 μM DCFH-DA, incubated at 37 °C for 30 min. Fluorescence intensity of DCF was analyzed using a flow cytometer (NovoCyte, Agilent) and fluorescent microscopy (Zeiss).

***Anti-neuroinflammation assay in vitro***

BV2 cells were seeded at 1.5 × 10⁴ cells/mL in 6-well plates and cultured overnight. Cells were pretreated with nanozymes (25 or 50 μg/mL, 6 h), followed by stimulation with LPS (1 μg/mL, 24 h) to establish an in vitro microglial neuroinflammation model. After treatment, cells were stained with CD86-PE antibody, and surface CD86 expression was analyzed by flow cytometry. Additionally, quantitative real-time PCR (qPCR) was performed to evaluate the mRNA expression of proinflammatory microglial markers (IL-1β, IL-6, iNOS, and TNF-α). Primer sequences are listed in Supplementary Table 1.

***Neuronal oxidative stress injury assay in vitro***

HT22 cells were seeded into 6-well plates at 1 × 10⁵ cells/mL and cultured overnight. Cells were pretreated with nanozymes (25 or 50 μg/mL, 6 h) and then exposed to H₂O₂ (0.1 mM, 2 h) to induce oxidative stress injury. Thereafter, cells were stained with propidium iodide (PI) according to the manufacturer’s protocol (BD Pharmingen, No. 556463), and the proportion of PI-positive cells was determined by flow cytometry.

***Animals and ethical approval***

Male C57BL/6J mice (6-8 weeks old) were obtained from Beijing Huafukang Biotechnology Co., Ltd. and maintained under specific pathogen-free (SPF) conditions with ad libitum access to food and water. All animal procedures complied with the Guide for the Care and Use of Laboratory Animals and were approved by the Animal Ethics Committee of the State Key Laboratory of Biotherapy, Sichuan University, Chengdu, China (approval number: 20250314030).

***Establishment and treatment of the ICH mouse model***

The ICH model was established as previously described[1] by stereotaxic injection of collagenase VII or autologous arterial blood into the striatum. Mice were anesthetized with 1% sodium pentobarbital (35 mg/kg, intraperitoneally) and placed in a stereotaxic apparatus once the righting reflex was abolished. Following scalp incision and exposure of the bregma, a burr hole (≈0.5 mm diameter) was drilled at 0.5 mm anterior and 2.0 mm lateral to the right of the bregma. The needle was advanced 3.5 mm beneath the skull surface to the injection site. Collagenase VII (0.5 μL at 0.1 μL/min) or autologous blood (30 μL at 3 μL/min) was delivered, the needle retained for 10 min, and then slowly withdrawn. The burr hole was sealed with bone wax, and the incision was sutured. Mice were placed on a 30 °C heating pad until recovery.

One hour after ICH induction, animals were randomly assigned to NS, Mn-ZIF, or Ru@Mn-ZIF nanozymes treatment groups. Based on a previous study[2], we selected a dose of 8 mg/kg for the nanozyme treatment in collagenase-induced ICH mice, administered via both intranasal instillation and intravenous injection. Intranasal dosing consisted of a total volume of 20 μL administered in two aliquots, whereas intravenous injection was delivered at a total volume of 100 μL. Treatments were given once daily for three consecutive days. Based on the observed therapeutic effects in the collagenase model, autologous blood–induced ICH mice were treated only via intranasal administration.

Therapeutic efficacy was assessed at days 1, 3, 5, and 7 post-ICH using the beam balance test, modified Garcia score, and circling test, as previously reported[3]. Body weight was recorded throughout the observation period.

***Histopathological assessment of brain tissue***

To evaluate the pathological impact of nanozymes treatment on ICH, blood–brain barrier (BBB) integrity was examined on day 3 post-ICH using Evans blue leakage assays, and hemoglobin content in brain tissue was quantified with Drabkin’s reagent [4]. Brain tissues were harvested at days 3 and 7 following behavioral assessments, fixed in 4% paraformaldehyde, embedded in paraffin, and sectioned. Histological analyses were performed using hematoxylin–eosin (HE), Nissl, and Perls’ Prussian blue staining according to the manufacturer’s protocols.

Immunohistochemistry was performed to determine the perihematomal expression of IL-1β, IL-6, and TNF-α. Immunofluorescence staining was used to detect cellular oxidative damage marker 8-OHdG, microglial marker Iba-1, astrocytic marker GFAP, and neutrophil marker MPO.

***Biosafety assessment of nanozymes treatment***

On day 7 post-ICH, blood was collected from the retro-orbital sinus under anesthesia following behavioral testing. Mice were subsequently euthanized, and major organs (heart, liver, spleen, lung, and kidney) were harvested for HE staining. Blood samples were allowed to clot overnight at 4 °C, centrifuged, and serum was collected for biochemical assays.

***Statistical analysis***

All data are expressed as mean ± SEM. Statistical analyses were conducted using GraphPad Prism 10.0. Group comparisons were performed using one-way ANOVA, two-way ANOVA, or Student’s t-test, and correlations were evaluated by Spearman’s correlation coefficient. A p-value < 0.05 was considered statistically significant (*p < 0.05, **p < 0.01, ***p < 0.001, ****p < 0.0001).

**Supplementary Figures**


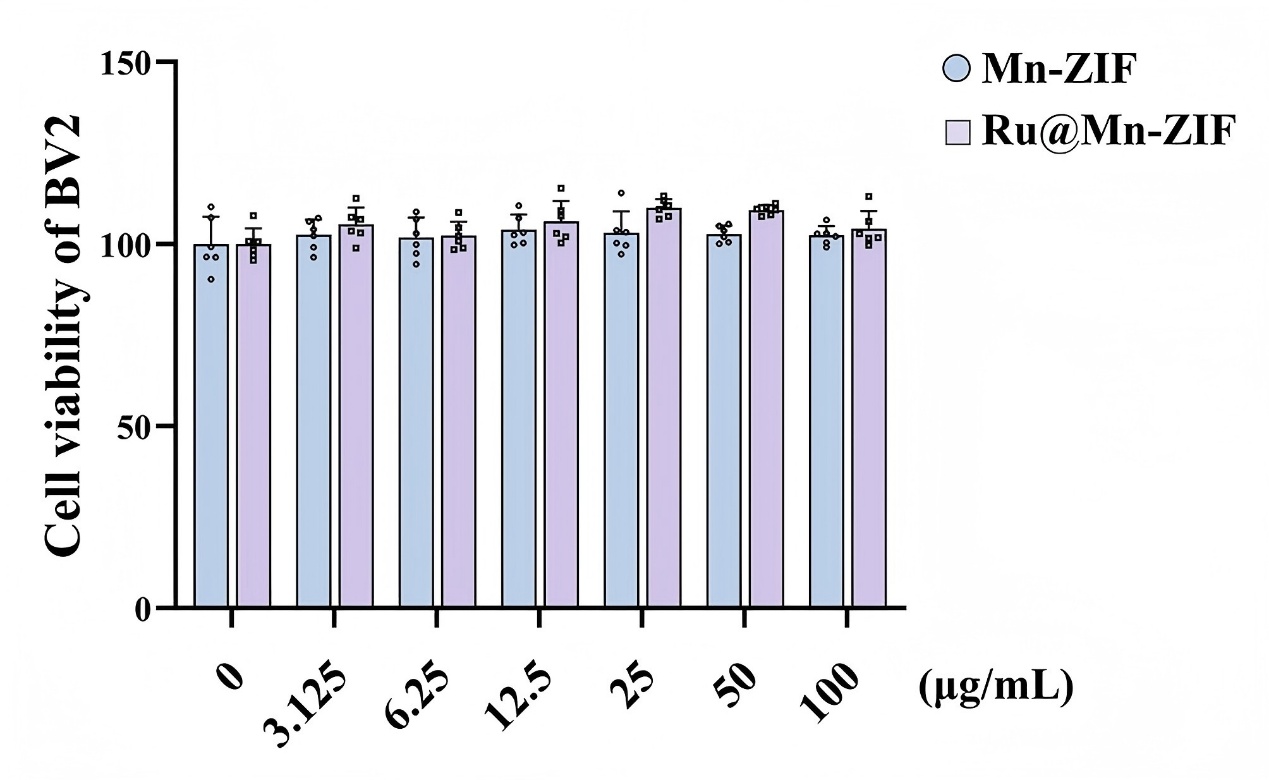


**Figure S1.** BV2 Cell viability assay under different concentrations of Mn-ZIF or Ru@Mn-ZIF nanozyme.


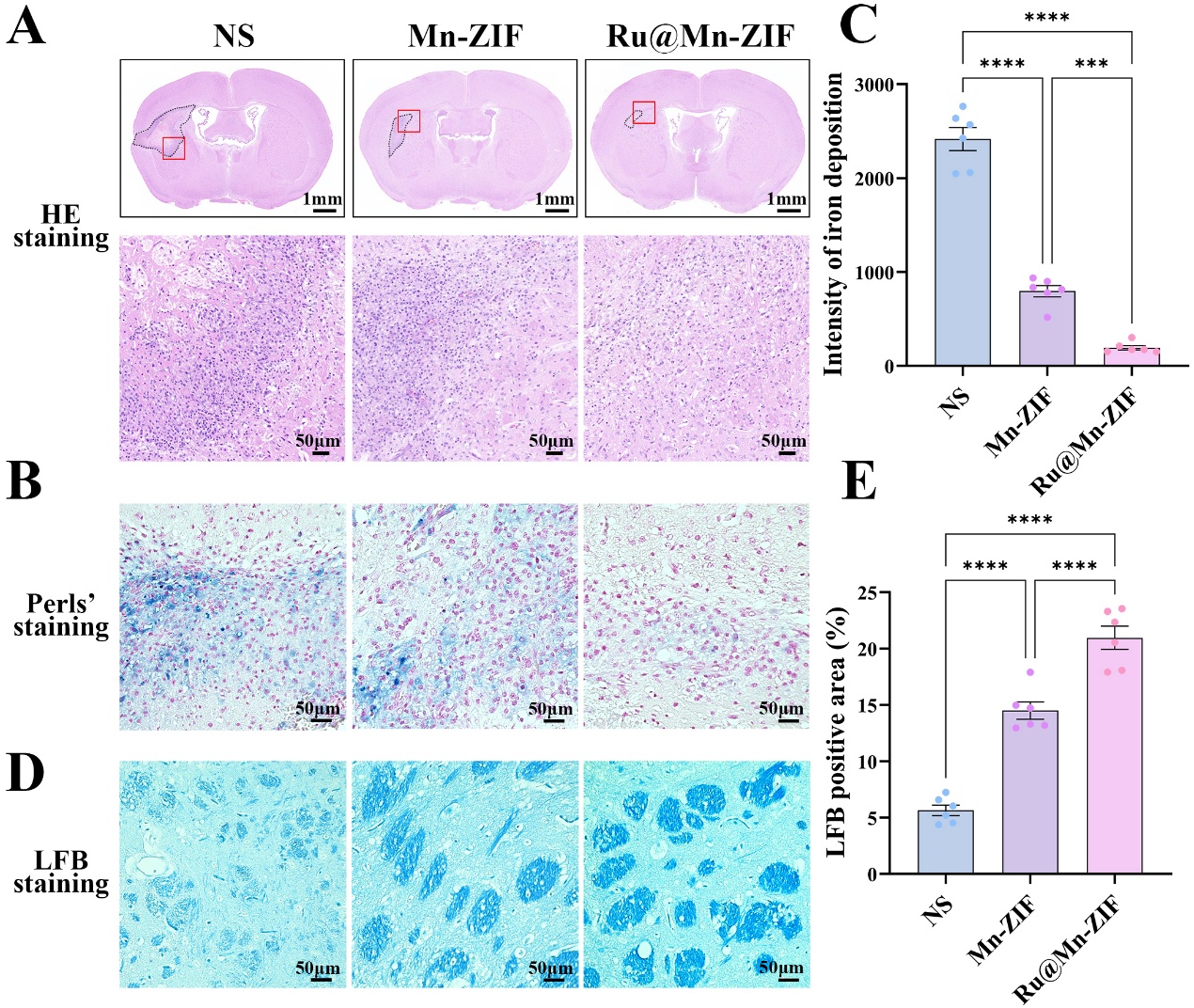


**Figure S2.** Histopathological evaluation of brain tissue in collagenase-induced ICH mice after 7 days of intranasal nanozyme treatment. A) HE staining showing reduced glial scar formation in the perihematomal region on day 7 following intranasal nanozyme administration (upper panel, 2× magnification; lower panel, 40× magnification). Scale bars: 1 mm (upper), 50 μm (lower). B, C) Perls’ Prussian blue staining demonstrating significantly decreased iron deposition in perihematomal regions after intranasal nanozyme treatment (n = 6). Scale bars: 50 μm. D, E) Luxol fast blue (LFB) staining showing markedly reduced myelin loss in perihematomal regions after intranasal nanozyme treatment (n = 6). Scale bars: 50 μm. Data are presented as mean ± SEM and analyzed by one-way ANOVA followed by Tukey's post-hoc test. ***p < 0.001, ****p < 0.0001.


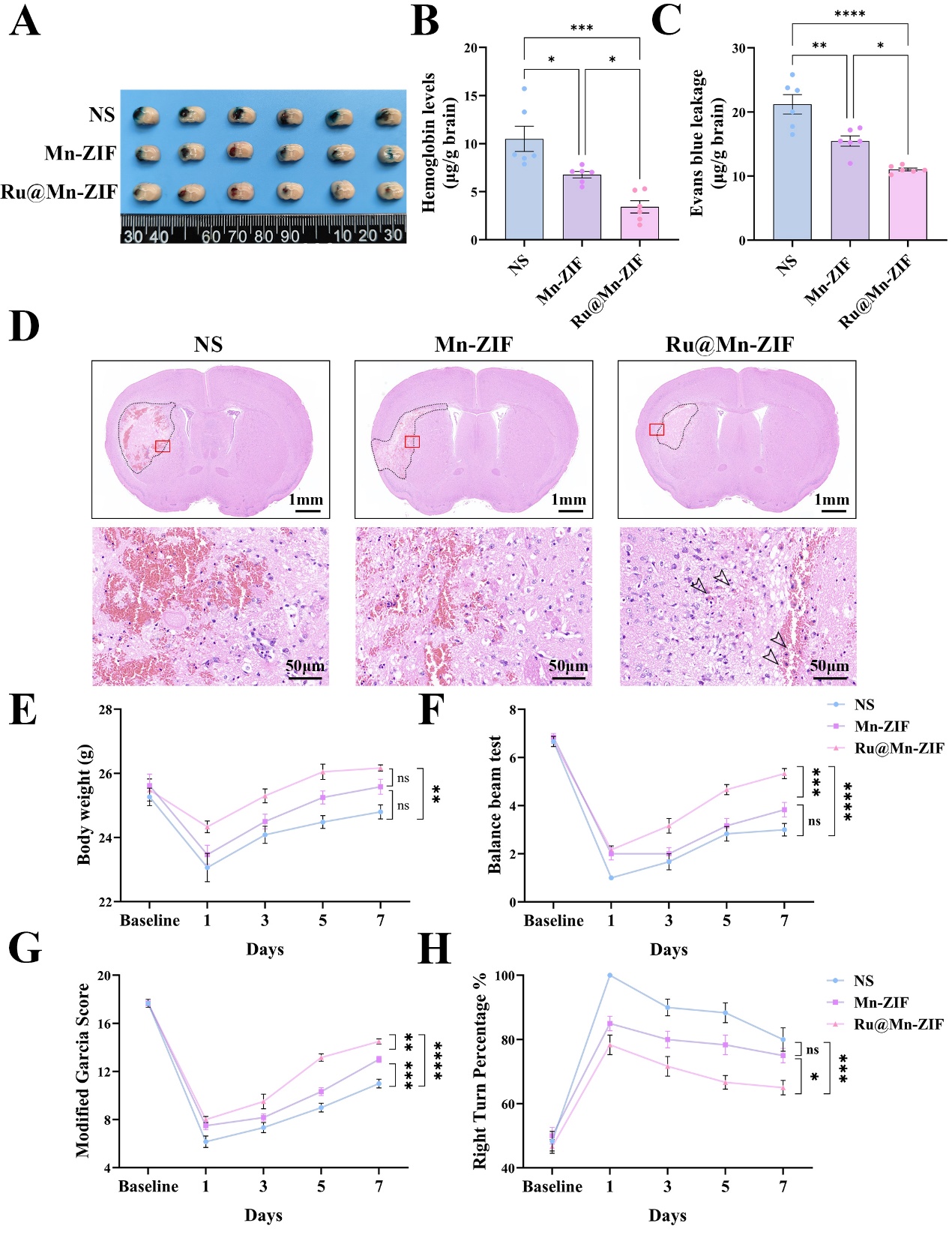


**Figure S3.** Therapeutic evaluation of intravenously administered nanozymes in collagenase-induced ICH mice. A–C) Intravenous nanozyme treatment significantly reduced hematoma volume and Evans blue extravasation in the brain 3 days post-administration (n = 6). Data are presented as mean ± SEM and analyzed using one-way ANOVA followed by Tukey's post-hoc test. *p < 0.05, **p < 0.01, ***p < 0.001, ****p < 0.0001. D) HE staining showing reduced perihematomal edema and inflammatory cell infiltration on day 3 after intravenous nanozyme treatment. In the Ru@Mn-ZIF group, numerous phagocytes containing engulfed red blood cells were observed (arrows). Upper panel, 2× magnification; lower panel, 40× magnification. Scale bars: 1 mm (upper), 50 μm (lower). E) Intravenous Ru@Mn-ZIF nanozyme administration significantly reversed body weight loss in ICH mice (n = 6). F, G) Intravenous nanozyme treatment significantly improved neurological performance as reflected by increased balance beam test scores and modified Garcia scores (n = 6). H) Intravenous Ru@Mn-ZIF nanozyme treatment significantly reduced the percentage of right turns in the circling test (n = 6). Data are presented as mean ± SEM and analyzed using two-way ANOVA followed by Tukey's post-hoc test. *p < 0.05, **p < 0.01, ***p < 0.001, ****p < 0.0001.


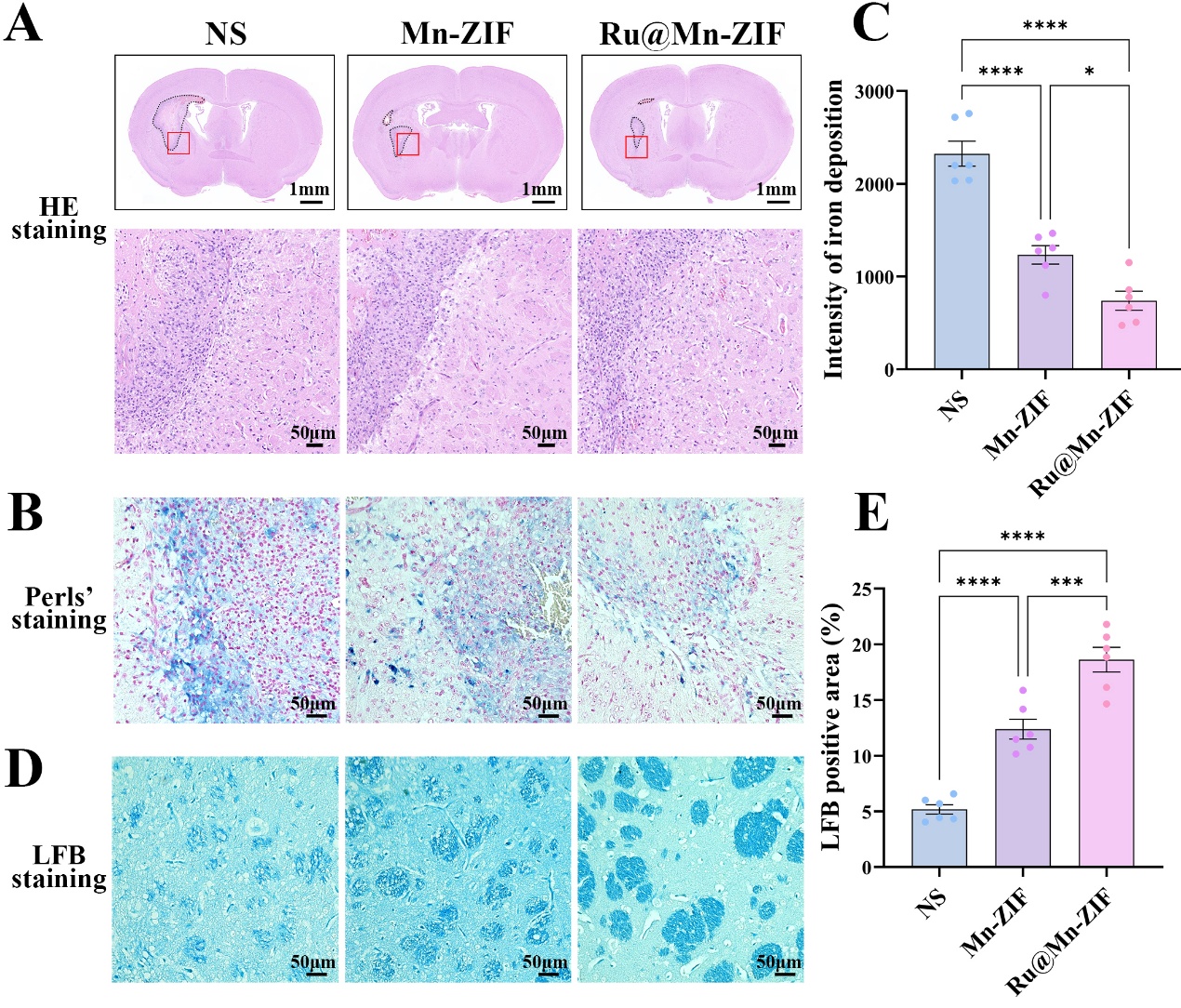


**Figure S4.** Histopathological evaluation of brain tissue in collagenase-induced ICH mice after 7 days of intravenous nanozyme treatment. A) HE staining showing reduced glial scar formation in perihematomal regions on day 7 following intravenous nanozyme administration (upper panel, 2× magnification; lower panel, 40× magnification). Scale bars: 1 mm (upper), 20 μm (lower). B, C) Perls’ Prussian blue staining demonstrating significantly decreased iron deposition in perihematomal regions after intravenous nanozyme treatment (n = 6). Scale bars: 50 μm. D, E) Luxol fast blue (LFB) staining showing markedly reduced myelin loss in perihematomal regions after intravenous nanozyme treatment (n = 6). Scale bars: 50 μm. Data are presented as mean ± SEM and analyzed using one-way ANOVA followed by Tukey's post-hoc test. *p < 0.05, ***p < 0.001, ****p < 0.0001.


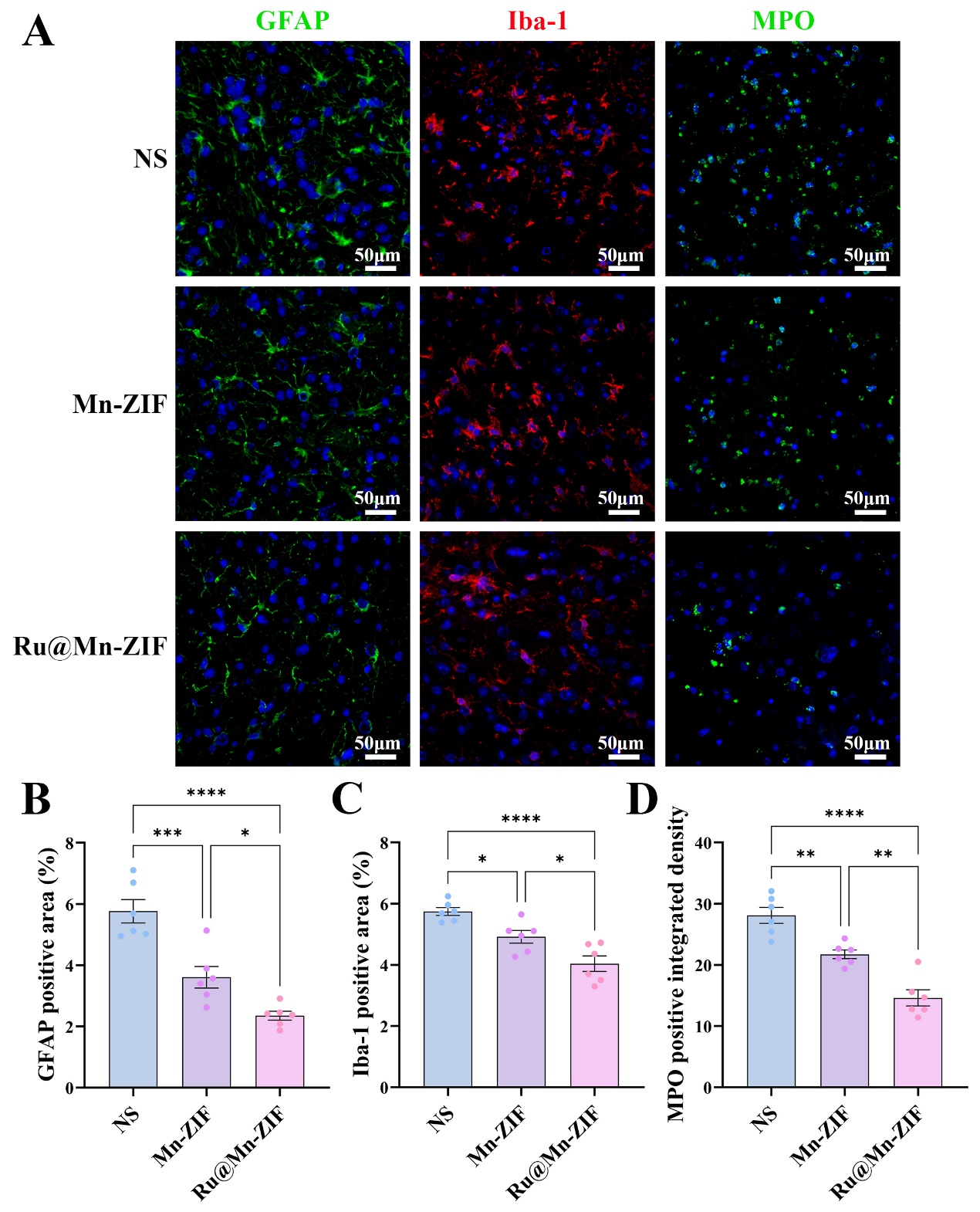


**Figure S5.** Inflammatory cell activation in the perihematomal region of ICH mice on day 3 following intravenous treatment. A) Immunofluorescence staining of major inflammatory cells in perihematomal brain tissue: astrocytes (GFAP), microglia/macrophages (Iba-1), and neutrophils (MPO). Scale bars: 50 μm. B) Quantification of GFAP-positive astrocytic area (n = 6). C) Quantification of Iba-1–positive microglial/macrophage area (n = 6). D) Quantification of MPO-positive neutrophil fluorescence intensity (n = 6). Data are presented as mean ± SEM and analyzed by one-way ANOVA followed by Tukey's post-hoc test. *p < 0.05, **p < 0.01, ***p < 0.001, ****p < 0.0001.


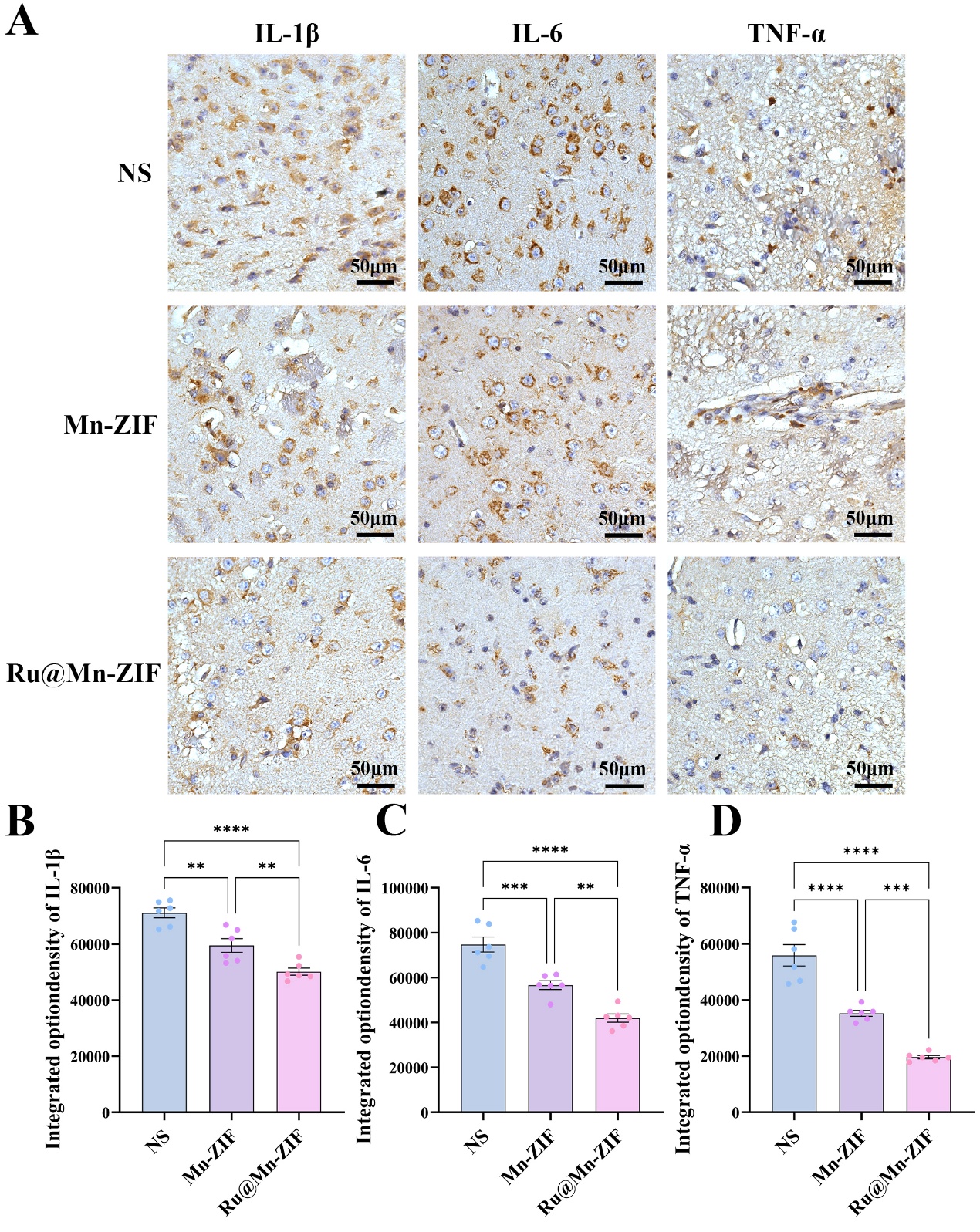


**Figure S6.** Expression of proinflammatory cytokines in the perihematomal region of ICH mice on day 3 following intravenous treatment. A) Immunohistochemical staining of major proinflammatory cytokines in perihematomal brain tissue. Scale bars: 50 μm. B) Quantification of IL-1β immunohistochemical DAB integrated optical density (IOD) (n = 6). C) Quantification of IL-6 immunohistochemical DAB IOD (n = 6). D) Quantification of TNF-α immunohistochemical DAB IOD (n = 6). Data are presented as mean ± SEM and analyzed by one-way ANOVA followed by Tukey's post-hoc test. **p < 0.01, ***p < 0.001, ****p < 0.0001.


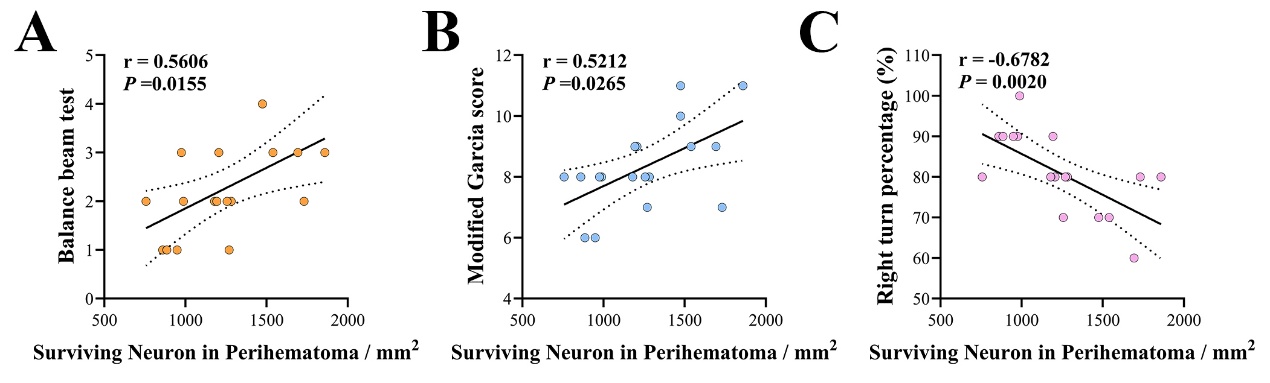


**Figure S7.** Correlation analysis between perihematomal neuronal density and neurological outcomes in collagenase-induced ICH mice after 3 days of intranasal nanozyme treatment. Perihematomal neuronal density showed a significant positive correlation with beam balance test scores and modified Garcia scores, and a significant negative correlation with the percentage of right turns.


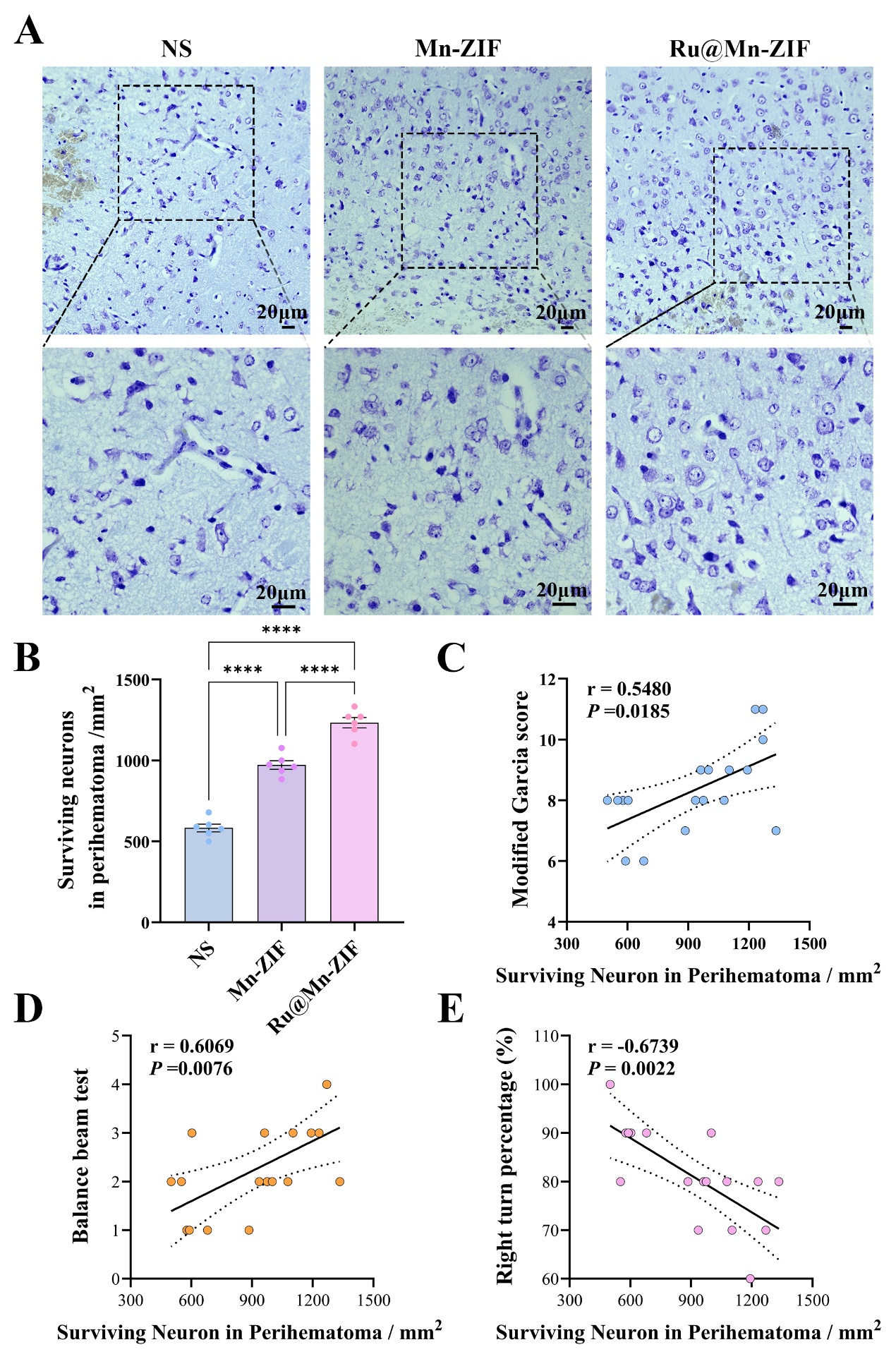


**Figure S8.** Correlation analysis between perihematomal neuronal density and neurological outcomes in collagenase-induced ICH mice after 3 days of intravenous nanozyme treatment. A) Nissl staining of perihematomal brain tissue (upper panel, 20× magnification; lower panel, 40× magnification). Scale bars: 20 μm. B) Quantification of perihematomal surviving neuronal density based on Nissl staining (n = 6). Data are presented as mean ± SEM and analyzed using one-way ANOVA followed by Tukey's post-hoc test. ****p < 0.0001. C–E) Correlation analysis showing that perihematomal neuronal density was significantly positively correlated with beam balance test scores and modified Garcia scores, and significantly negatively correlated with the percentage of right turns.


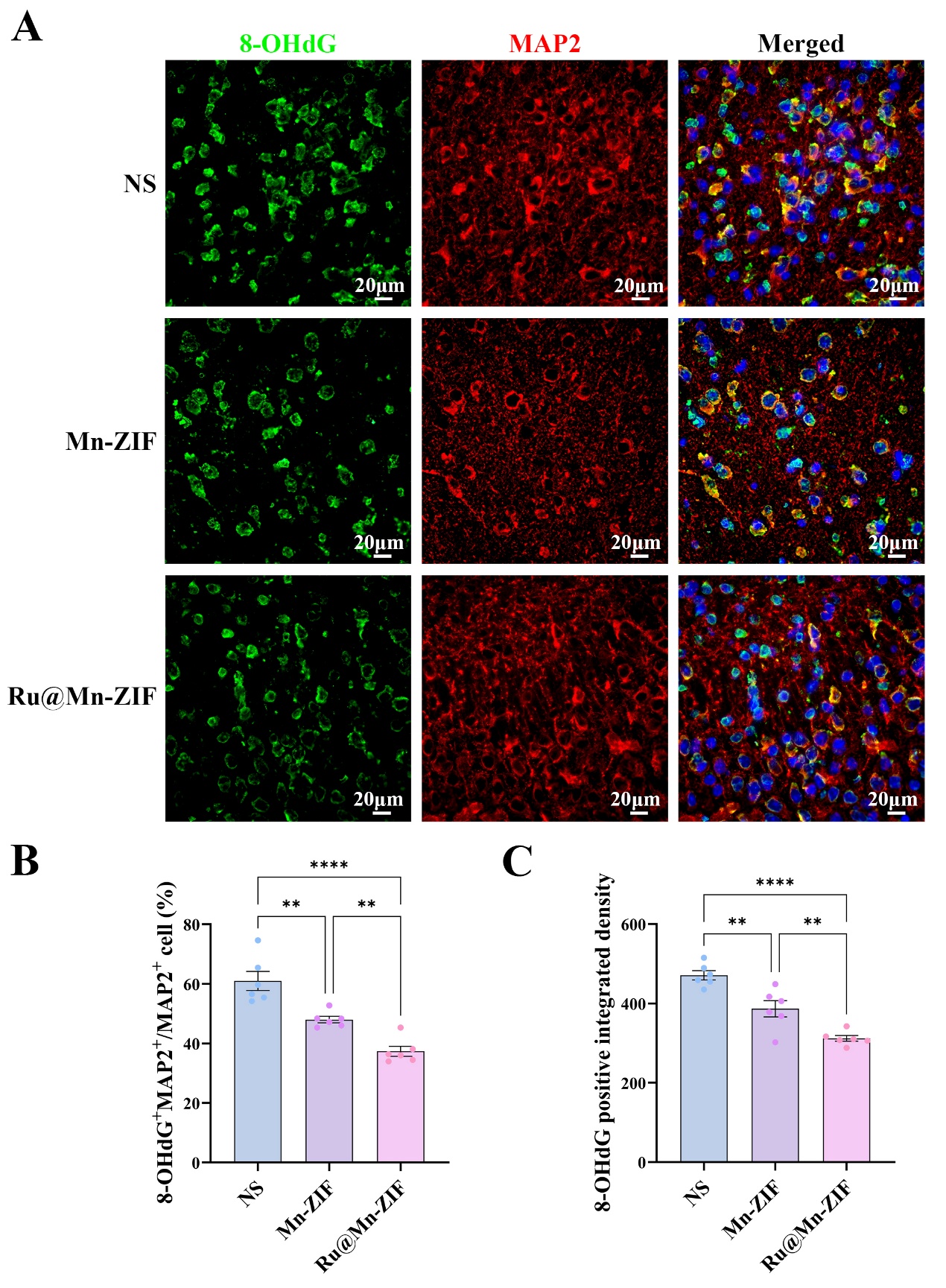


**Figure S9.** Evaluation of neuronal DNA oxidative stress injury in perihematomal regions of collagenase-induced ICH mice after 3 days of intravenous nanozyme treatment. A) Immunofluorescence staining of neuronal DNA oxidative stress in perihematomal regions: 8-OHdG (green) and MAP2 (red). Scale bars: 50 μm. B) Percentage of 8-OHdG–positive neurons among total neurons in the perihematomal region (n = 6). C) Quantification of 8-OHdG–positive fluorescence intensity in perihematomal regions (n = 6). Data are presented as mean ± SEM and analyzed by one-way ANOVA followed by Tukey's post-hoc test. **p < 0.01, ****p < 0.0001.


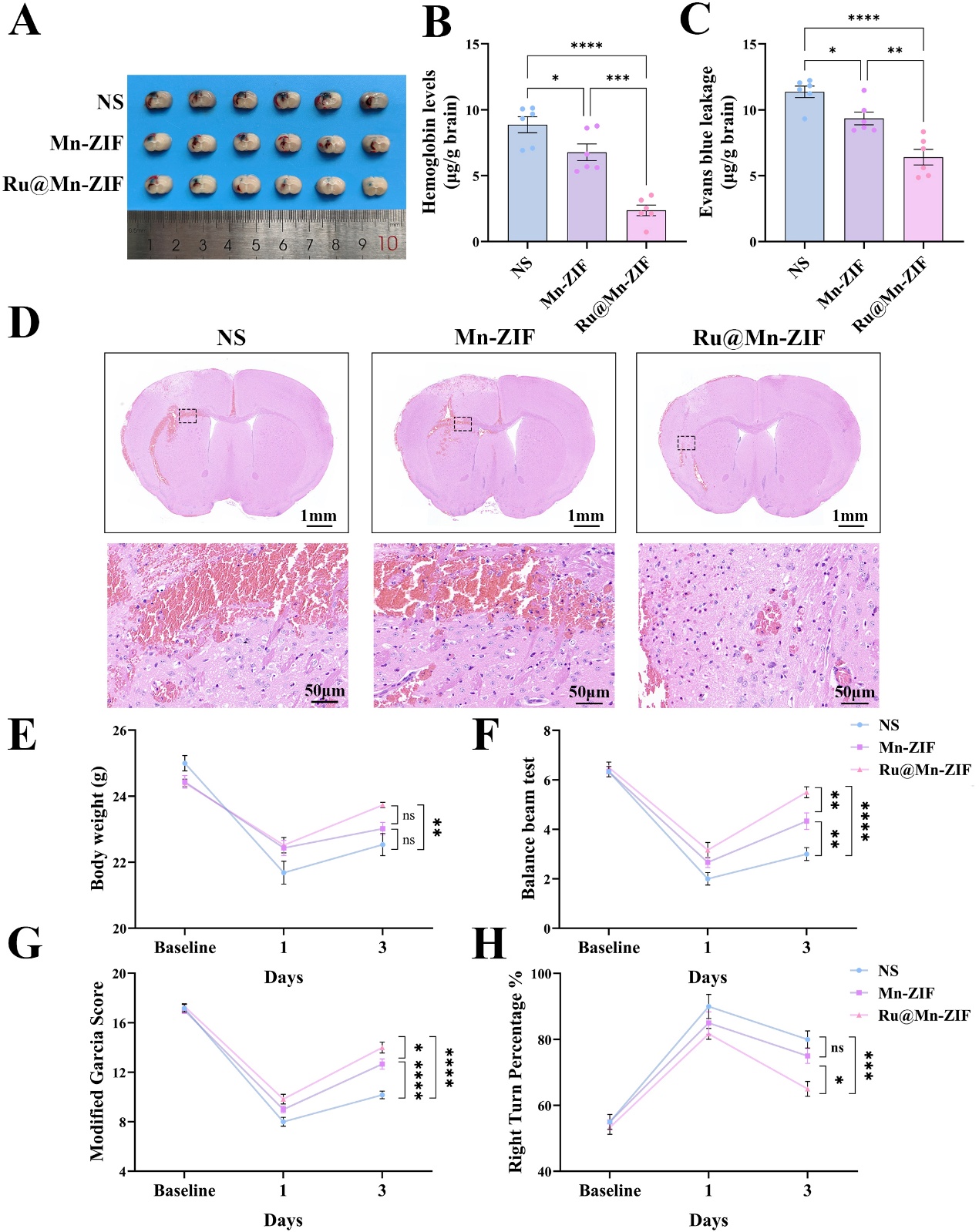


**Figure S10.** Therapeutic evaluation of intranasally administered nanozymes in autologous blood–induced ICH mice. A–C) Intranasal nanozyme treatment significantly reduced hematoma volume and Evans blue extravasation in the brain 3 days post-administration (n = 6). Data are presented as mean ± SEM and analyzed using one-way ANOVA followed by Tukey's post-hoc test. *p < 0.05, **p < 0.01, ***p < 0.001, ****p < 0.0001. D) HE staining showing reduced perihematomal edema and inflammatory cell infiltration on day 3 after nanozyme treatment. In the Ru@Mn-ZIF group, numerous phagocytes containing engulfed red blood cells were observed. Upper panel, 2× magnification; lower panel, 40× magnification. Scale bars: 1 mm (upper), 50 μm (lower). E) Intravenous Ru@Mn-ZIF nanozyme administration significantly reversed body weight loss in ICH mice (n = 6). F,G) Nanozyme treatment significantly improved neurological performance as reflected by increased balance beam test scores and modified Garcia scores (n = 6). H) Intravenous Ru@Mn-ZIF nanozyme treatment significantly reduced the percentage of right turns in the circling test (n = 6). Data are presented as mean ± SEM and analyzed using two-way ANOVA followed by Tukey's post-hoc test. *p < 0.05, **p < 0.01, ***p < 0.001, ****p < 0.0001.


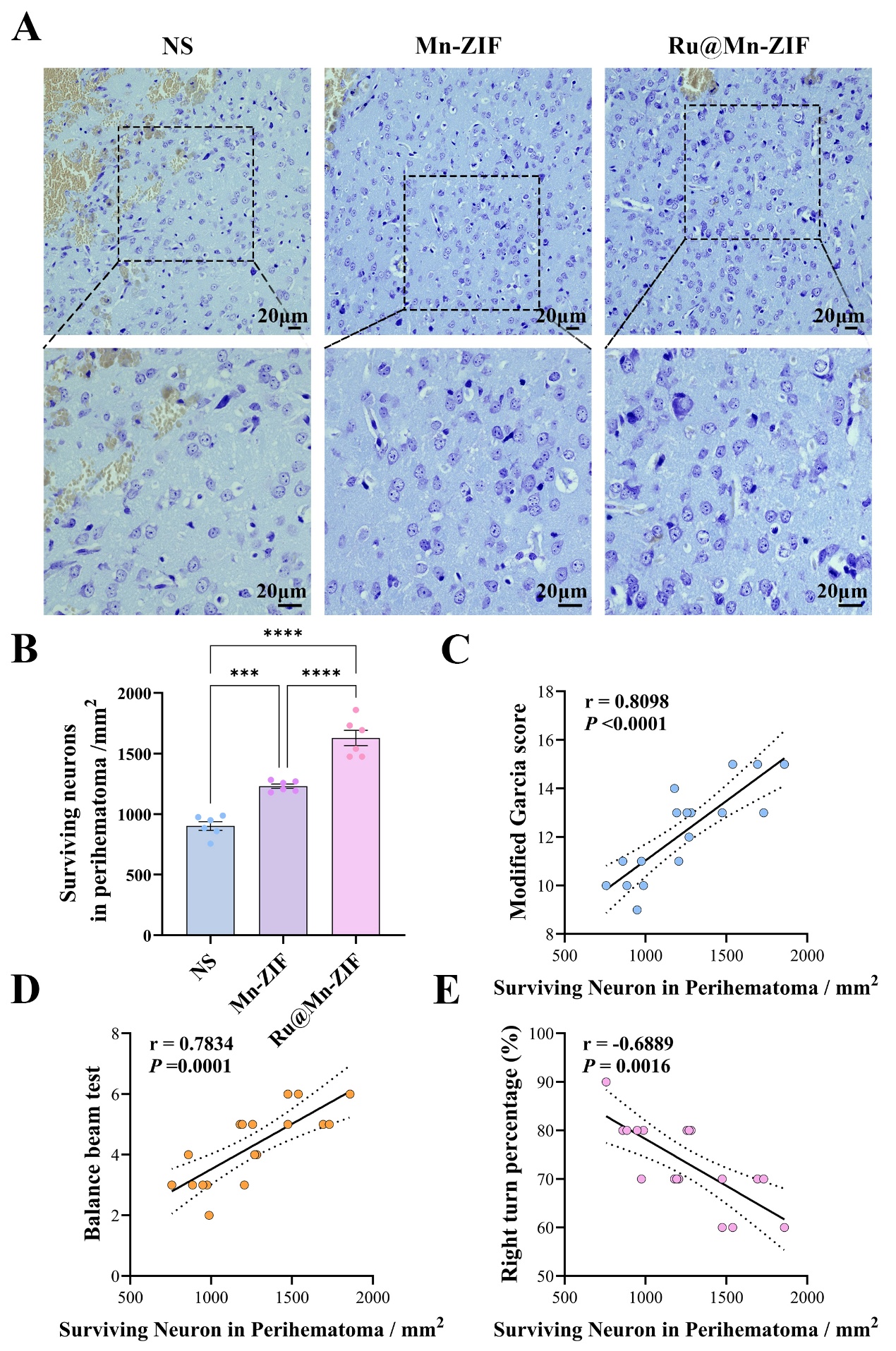


**Figure S11.** Correlation analysis between perihematomal neuronal density and neurological outcomes in autologous blood–induced ICH mice after 3 days of intranasal nanozyme treatment. A) Nissl staining of perihematomal brain tissue (upper panel, 20× magnification; lower panel, 40× magnification). Scale bars: 20 μm. B) Quantification of surviving neuronal density in perihematomal regions based on Nissl staining (n = 6). Data are presented as mean ± SEM and analyzed using one-way ANOVA followed by Tukey's post-hoc test. ****p < 0.0001. C–E) Correlation analysis showing that perihematomal neuronal density was significantly positively correlated with beam balance test scores and modified Garcia scores, and significantly negatively correlated with the percentage of right turns.


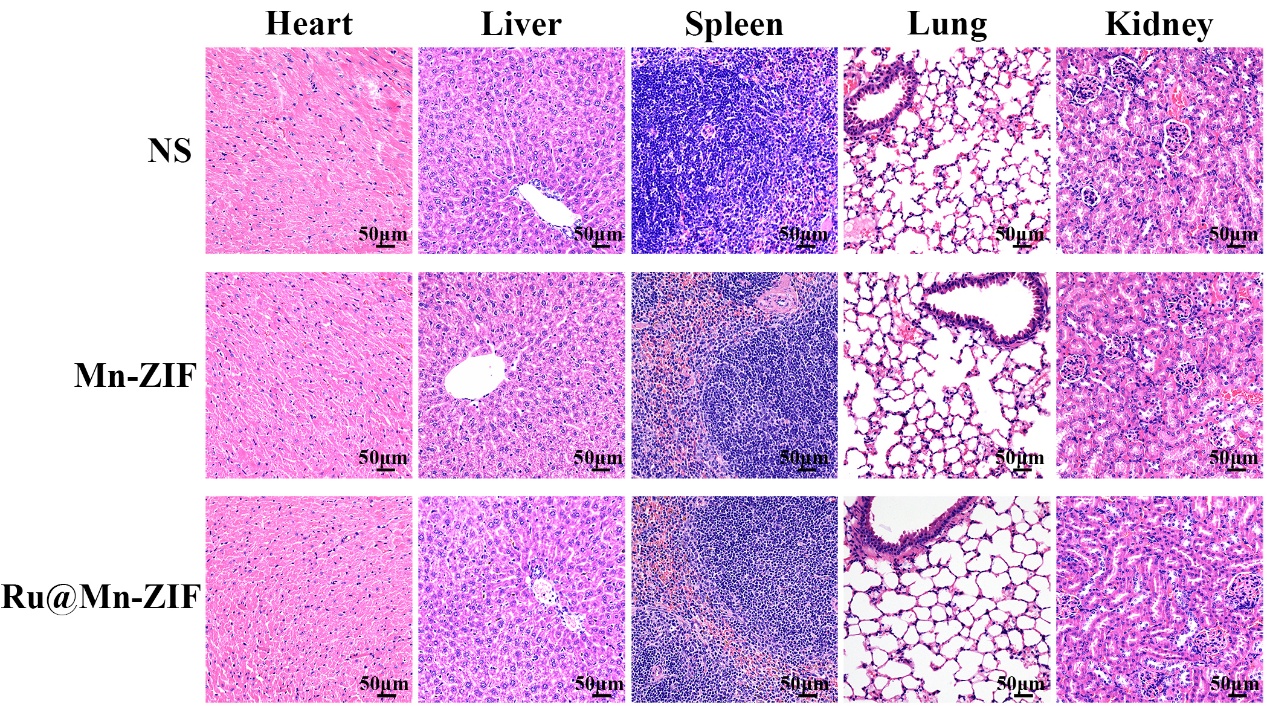


**Figure S12.** Toxicity evaluation of major organs in ICH mice after intranasal nanozyme treatment by H&E staining. Representative H&E staining images of major organs collected from ICH mice following intranasal nanozyme administration. Scale bars: 50 μm.


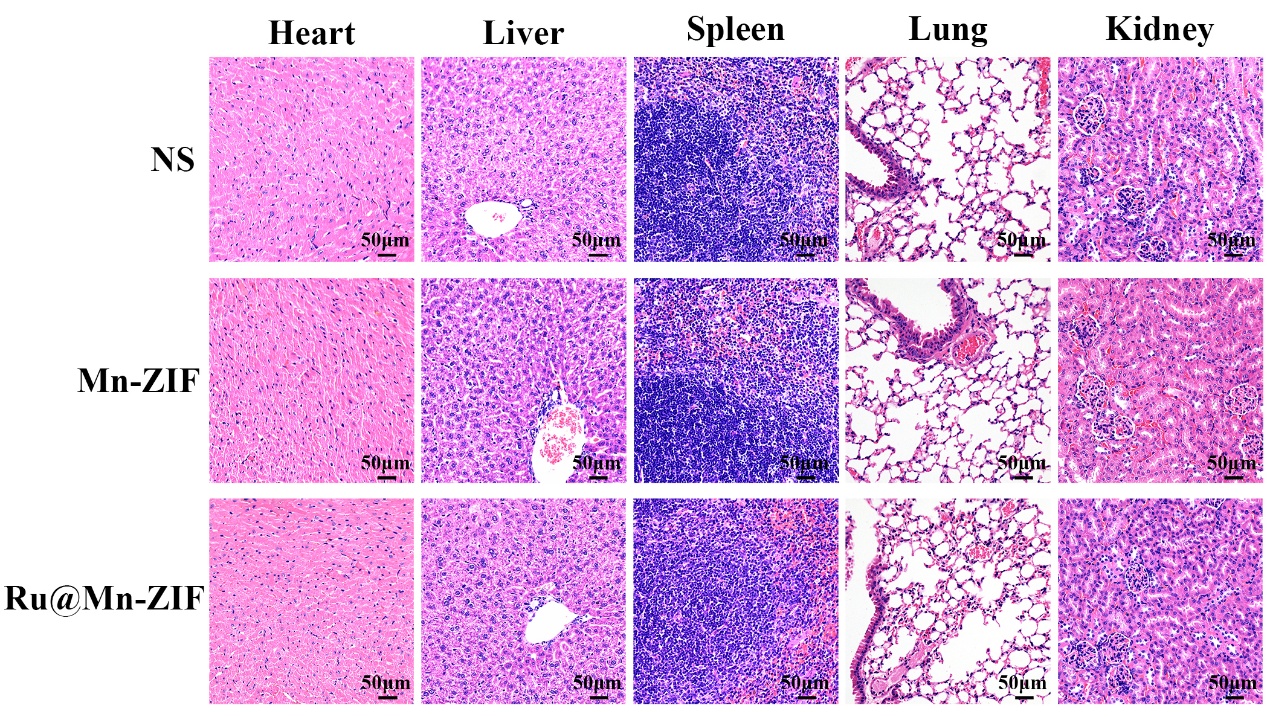


**Figure S13.** Toxicity evaluation of major organs in ICH mice after intravenous nanozyme treatment by H&E staining. Representative H&E staining images of major organs collected from ICH mice following intravenous nanozyme administration. Scale bars: 50 μm.


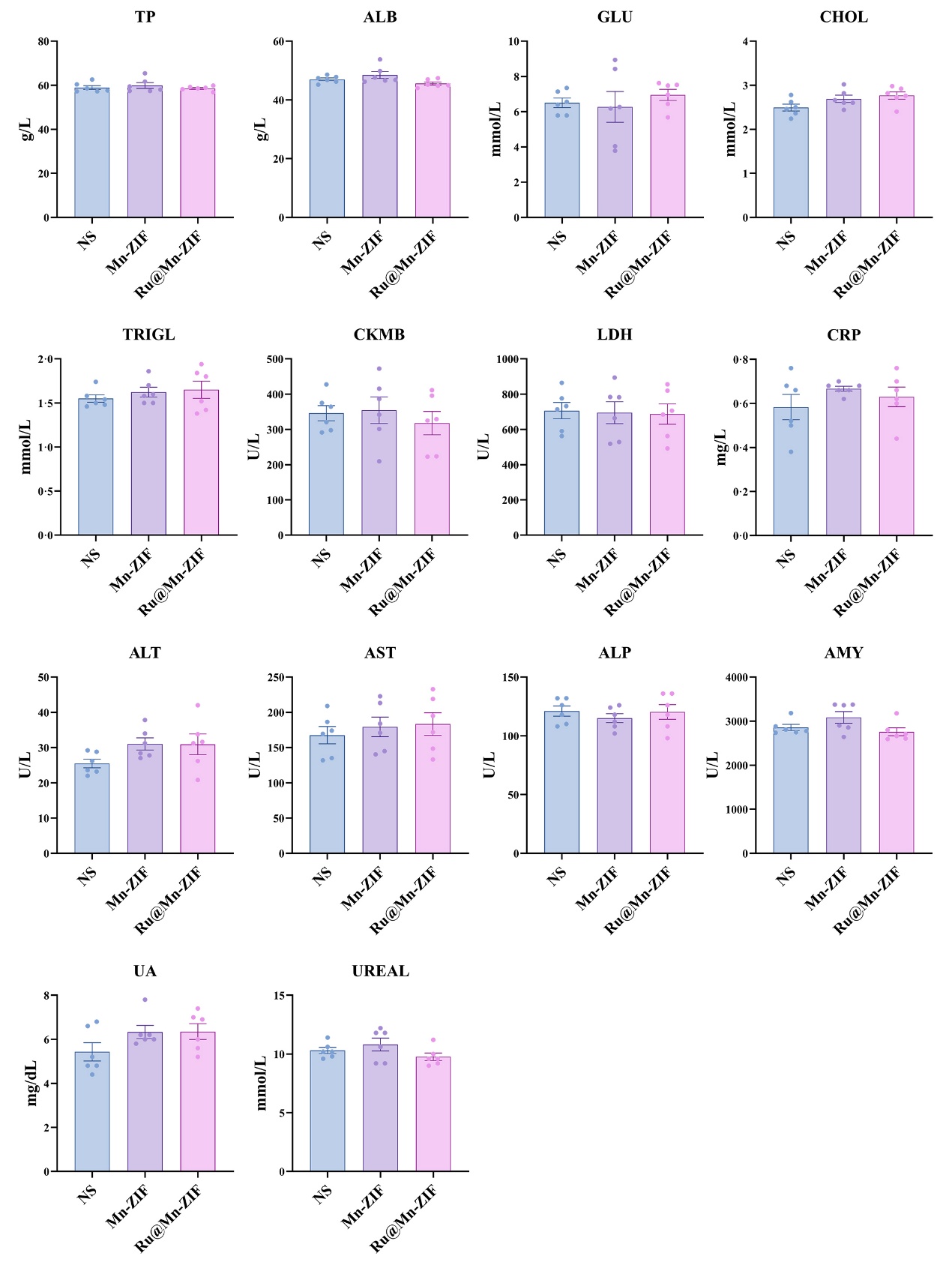


**Figure S14.** Toxicity evaluation of ICH mice by serum biochemical analysis after 7 days of intranasal nanozyme administration (n = 6).


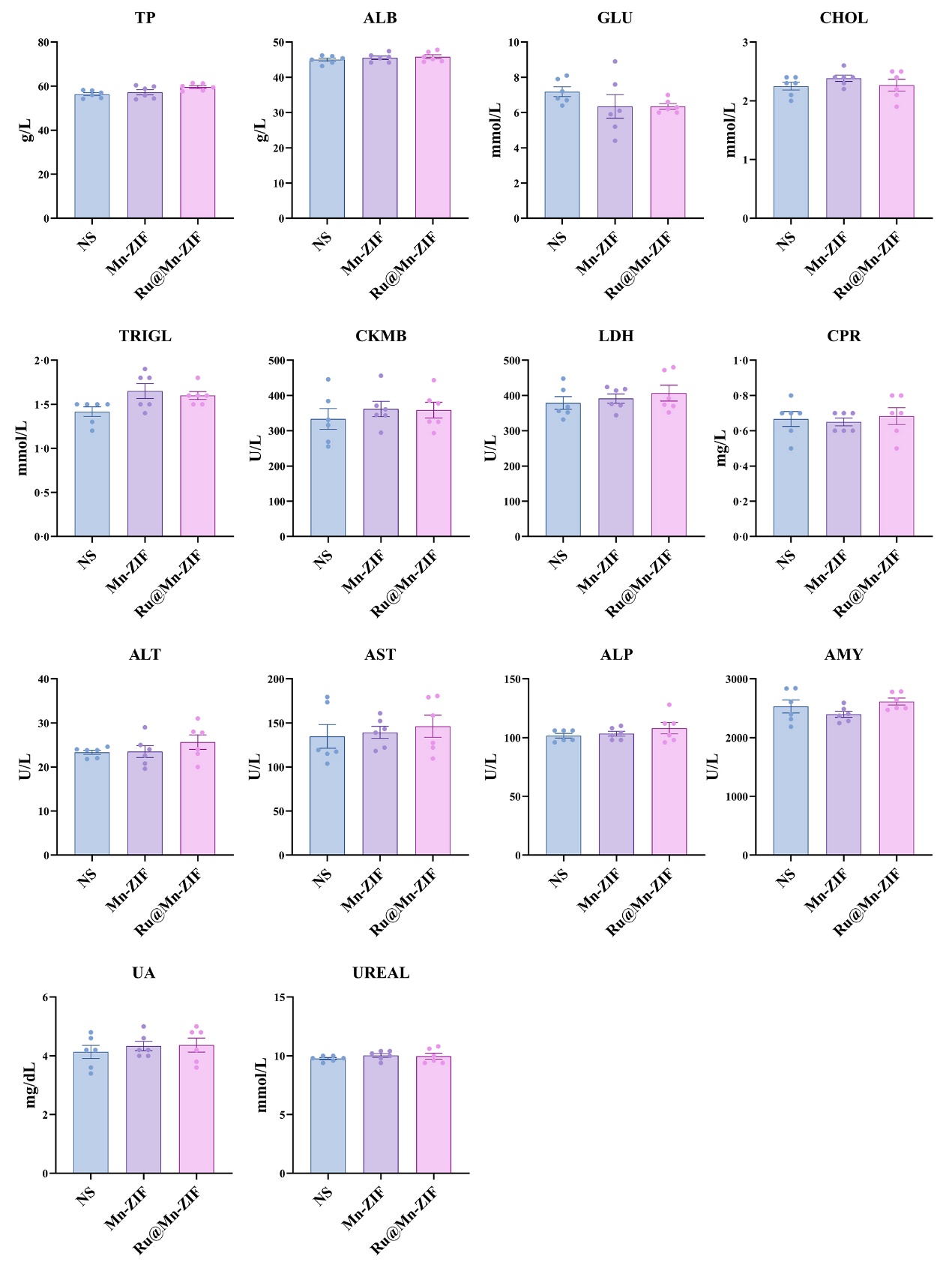


**Figure S15.** Toxicity evaluation of ICH mice by serum biochemical analysis after 7 days of intravenous nanozyme administration (n = 6).

**Supplementary Table**

**Table 1 the primer sequences of Real-Time qPCR assay**

| Species | Gene |  | Primer sequence |
| --- | --- | --- | --- |
| Mouse | GAPDH | Forward | AGGTCGGTGTGAACGGATTTG |
| Mouse | GAPDH | Reverse | TGTAGACCATGTAGTTGAGGTCA |
| Mouse | iNOS | Forward | GTTCTCAGCCCAACAATACAAGA |
| Mouse | iNOS | Reverse | GTGGACGGGTCGATGTCAC |
| Mouse | TNF-α | Forward | CCCTCACACTCAGATCATCTTCT |
| Mouse | TNF-α | Reverse | GCTACGACGTGGGCTACAG |
| Mouse | IL-6 | Forward | TAGTCCTTCCTACCCCAATTTCC |
| Mouse | IL-6 | Reverse | TTGGTCCTTAGCCACTCCTTC |
| Mouse | IL-1β | Forward | TACCTGTGTCTTTCCCGTGG |
| Mouse | IL-β | Reverse | GTTGTTCATCTCGGAGCCTGT |

**References**

[1] P. R. Krafft, W. B. Rolland, K. Duris, T. Lekic, A. Campbell, J. Tang, J. H. Zhang, *J Vis Exp* **2012**, (67), e4289, <https://doi.org/10.3791/4289>.

[2] Q. Bai, Y. Han, S. Khan, T. Wu, Y. Yang, Y. Wang, H. Tang, Q. Li, W. Jiang, *Adv. Healthc. Mater.* **2024**, *13* (2), e2302526, <https://doi.org/10.1002/adhm.202302526>.

[3] M. Balkaya, J. M. Kröber, A. Rex, M. Endres, *J. Cereb. Blood Flow Metab.* **2013**, *33* (3), 330, <https://doi.org/10.1038/jcbfm.2012.185>.

[4] Z. Duan, W. Zhou, S. He, W. Wang, H. Huang, L. Yi, R. Zhang, J. Chen, X. Zan, C. You, X. Gao, *Small Methods* **2024**, *8* (12), e2400304, <https://doi.org/10.1002/smtd.202400304>.
